# Evaluation of Bioactive Compounds and Antioxidant Activity in 51 Minor Tropical Fruits of Ecuador

**DOI:** 10.3390/foods12244439

**Published:** 2023-12-11

**Authors:** Elena Coyago-Cruz, Aida Guachamin, Michael Villacís, Jason Rivera, María Neto, Gabriela Méndez, Jorge Heredia-Moya, Edwin Vera

**Affiliations:** 1 Carrera de Ingeniería en Biotecnología de los Recursos Naturales, Universidad Politécnica Salesiana, Sede Quito, Campus El Girón, Av. 12 de Octubre N2422 y Wilson, Quito 170143, Ecuador; 2 Centro de Investigación Biomédica (CENBIO), Facultad de Ciencias de la Salud Eugenio Espejo, Universidad UTE, Quito 170527, Ecuador; 3 Escuela Politécnica Nacional, Departamento de Ciencias de los Alimentos y Biotecnología, Facultad de Ingeniería Química, Av. 12 de Octubre N2422 y Veintimilla, Quito 170524, Ecuador

**Keywords:** bioactive compound, functional foods, carotenoids, phenolics, antioxidant activity

## Abstract

Less common tropical fruits have been the subject of little research, leaving a vast field to be explored. In this context, a comprehensive study was carried out on the bioactive compounds and antioxidant capacity of 51 non-traditional fruits consumed in Ecuador. Vitamin C, organic acids, carotenoids, and phenolic compounds were evaluated using microextraction and rapid resolution liquid chromatography (RRLC) techniques, while antioxidant activity was measured using microplate readings. The results showed high levels of vitamin C (768.2 mg/100 g DW) in *Dovyalis hebecarpa*, total organic acids (37.2 g/100 g DW) in *Passiflora tripartita*, carotenoids (487.0 mg/100 g DW) in *Momordica charantia*, phenolic compounds (535.4 mg/g DW) in *Nephelium lappaceum*, *Pourouma cecropiifolia* (161.4 µmol TE/g DW) and *Morus alba* (80.5 µmol AAE/g DW) in antioxidant activity. Effective extraction of carotenoids was also observed using a mixture of methanol: acetone: dichloromethane (1:1:2) with an extraction time of 2 min, while an 80% solution of 0.1% acidified methanol with hydrochloric acid with an extraction time of 3 min was highly effective for phenolics in fruit. These results provide a valuable basis for optimising future extraction processes of bioactive compounds from non-traditional fruits, with significant implications for their potential use in various nutritional and pharmaceutical contexts.

## 1. Introduction

Tropical fruits, often referred to as exotic or unconventional fruits, are not widely consumed in some areas of the world or are not part of the regular diet of a particular population. Only a tiny percentage of these fruits reach international markets; many still need to be discovered and are under-consumed. In the regions where they are produced, under-consumed tropical fruits play a crucial role in food and nutrition security and are a source of income for local producers. They account for about 75% of the total income of small rural households [1,2,3].

Tropical fruits generally originate from warm, tropical regions where climatic conditions favour their cultivation and development. They can be divided into three categories according to the extent of cultivation, production volume, and market demand: primary or major, and minor (secondary, and wild). Primary fruits include avocado (*Persea americana*), mango (*Mangifera indica*), pineapple (*Ananas comosus*), papaya (*Carica papaya*), and banana (*Musa paradisiaca*) [4]. In contrast, the remaining fruits are classified as secondary or minor (e.g., kiwi, carambola, tree tomato, bay berry, silverberry, passion fruit, guava, pitaya, jackfruit, etc.) and lesser-known as wild [5]. Tropical fruit production comes mainly from developing countries, especially Latin America and the Caribbean. Among these countries, Brazil, Colombia, and Ecuador are the leading producers of these fruits, with approximately 50% of production going to the fresh fruit market [4,6,7]. In this context, Ecuador stands out as a megadiverse country with a remarkable natural wealth that harbours various fruit species, some indigenous and others introduced. The indigenous population has domesticated these species over the years and now grows naturally in the region. However, many of these fruits are poorly known and under-exploited [8,9,10,11].

In recent years, the quest for a healthier lifestyle has led to increased consumption of fruit, vegetables, and functional foods. Diets rich in fruit and vegetables are positively associated with improved health [12,13,14,15]. In this context, fruits have attracted great interest due to their various compounds, such as polyphenols, flavonoids, carotenoids, vitamins C, A, E, folate, potassium, fibre, and others [8,16,17]. Fruit and vegetables contain bioactive compounds, which are products of the secondary metabolism of plants that have specific metabolic or physiological effects, and their concentrations depend on factors such as variety, growing conditions, storage, transport conditions, and industrialisation [17,18,19].

Both primary and secondary plant metabolites are essential for optimal body function and can help maintain a healthy immune system and overall health. In this regard, fruits have been shown to have a wide range of beneficial properties, including antioxidant, antiproliferative, anti-inflammatory, neuroprotective, antihypertensive, hypocholesterolemic, and hypoglycaemic. These effects can be used to prevent cardiovascular disease, diabetes, certain cancers, and neurodegenerative diseases [7,17,20].

The extent of the health benefits of eating lesser-known tropical fruits has yet to be fully understood. This is partly because they have been the subject of limited research by the scientific community. The search for new bioactive compound sources is intensifying, and non-traditional exotic fruits are attracting increasing interest in this area [1,8]. Although there are some reports on the composition and biological effects of certain traditional fruits consumed in Ecuador, the results are limited and need to provide a complete picture of the potential of these products as natural sources of bioactive compounds. Furthermore, studies have mainly focused on the profile of specific groups of bioactive compounds, such as vitamin C and polyphenols. While these compounds provide relevant information about the potential of some fruits, it is important to recognise that they are not the only components of importance, as other compounds are equally beneficial to health [8].

Obtaining and quantifying bioactive compounds in new species is challenging. Extraction depends on factors such as the technique, raw material, organic solvent, and other aspects. Conventional and non-conventional methods differ in terms of energy and solvent consumption. Traditional techniques are mainly influenced using organic solvents, temperatures, and stirring, as in the case of Soxhlet extraction, maceration, and hydrodistillation. Non-conventional or modern methods are more environmentally friendly due to their lower energy consumption and reduced use of organic solvents. These non-conventional techniques include supercritical extraction, pressurised fluid, ultrasound, microwaves, pulsed electric field, high voltage electric discharge, and high hydrostatic pressure and infrared spectroscopy. For example, ultrasound, a specific type of sound wave in the range of 20 kHz to 100 MHz, induces cavitation, which involves the formation, growth, and collapse of bubbles and enhances the release of compounds by disrupting plant cell walls; it is versatile and economically viable, making it an attractive option for the extraction of bioactive compounds [17,21,22,23].

In this context, some tropical fruits have been the subject of previous studies, but these have focused on specific aspects and have not provided a comprehensive assessment of different bioactive compounds. Although research has investigated compounds such as vitamin C, organic acids, carotenoids, and phenolics in some fruits such as *Bactris concinna*, *Miconia crocea*, *Passiflora mollisima*, *Prunus salicifolia*, and *Rubus niveus*. A recent literature review shows a lack of detailed studies integrating all these elements, as well as commercial quality and antioxidant activity, using microextractions, microplate readings, and chromatography [8,24]. Therefore, the lack of comprehensive research justifies the need to carry out the present study, with the aim of evaluating the commercial quality, bioactive compounds, and antioxidant capacity of fifty-one minor tropical fruits consumed in Ecuador. The results obtained will not only contribute to scientific knowledge but will also be of great benefit to small producers, opening prospects for the industrial application of certain fruits.

## 2. Materials and Methods

### 2.1. Reagents and Standards

Chemical compounds studied in this article: acetone (CAS 67-64-1) and trichloromethane (CAS 67-66-3) were of analytical grade, while acetonitrile (CAS 75-05-8), ethanol (CAS 64-17-5), ethyl acetate (CAS 141-78-6) and methanol (CAS 67-56-1) were of HPLC-grade and purchased from Fisher Chemical (Fischer Scientific Inc., Madrid, Spain). ABTS (2,2′-azino-bis-(3-ethylbenzothiazoline-6-sulphonic acid) (CAS 30931-67-0), *DL*-homocysteine (CAS 454-29-5), DPPH (2,2-Diphenyl-1-picrylhydrazyl) (CAS 1898-66-4), formic acid (CAS 64-18-6), metaphosphoric acid (CAS 37267-86-0), n-acetyl-*n*,*n*,*n*-trimethyl ammonium bromide (CAS 57-09-0), potassium persulfate (CAS 7727-21-1), monobasic potassium phosphate (CAS 7778-77-0) and sulfuric acid (CAS 7664-93-9) were of analytical grade and purchased from Sigma (Merck, Darmstadt, Germany). Hydrochloric acid (CAS7647-01-0) was of analytical grade and purchased from Labscan (RCI Labscan group, Dublin, Republic of Ireland). Water was purified using a NANOpureDiamondTM system (Barnsted Inc., Dubuque, IO, USA).

*L*-(+)-ascorbic acid 99.8% (CAS 50-81-7), citric acid 100.8% (CAS 77-92-9), malic acid 99.0% (CAS 97-67-6), *L*-(+)-tartaric acid 99.5% (CAS 87-69-4), caffeic acid 98.0% (CAS 331-39-5), chlorogenic acid 95.0% (CAS 327-97-9), chrysin 97.0% (CAS 480-40-0), *p*-coumaric acid 98.0% (CAS 501-98-4), *m*-coumaric acid 99.0% (CAS 588-30-7), *o*-coumaric acid 97.0% (CAS 614-60-8), ferulic acid 100.0% (CAS 1135-24-6), gallic acid 100.0% (CAS 149-91-7), *p*-hydroxybenzoic acid (CAS 99-96-7), 3-hydroxybenzoic acid 99.0% (CAS 99-06-3), 2,5-dihydroxybenzoic acid 98.0% (CAS 490-79-9), kaempferol 97.0% (CAS 520-18-3), luteolin 98% (CAS 491-70-3), naringin 95.0% (CAS 10236-47-2), quercetin 95.0% (CAS 849061-97-8), rutin 94.0% (CAS 153-18-4), shikimic acid 99.0% (CAS 138-59-0), syringic acid 95.0% (CAS 530-57-4), vanillic acid 97.0% (CAS 121-34-6), β-carotene 93.0% (CAS 7235-40-7), β-cryptoxanthin 97.0% (CAS 472-70-8), lutein (CAS 127-40-2), lycopene (CAS 502-65-8), zeaxanthin (CAS 144-68-3), and Trolox 98% (CAS 53188-07-1), were of standard grade and purchased from Sigma (Merck, Darmstadt, Germany).

### 2.2. Physico-Chemical Analyses

The study included ripe fruits from 21 different families and 51 exotic species purchased from various local markets in Ecuador during the months of January to March 2021 (Table 1). Samples were selected randomly, considering two portions of at least 10 units for large fruits (total of 20 fruits), 2 kg for medium (total of 60 fruits), and 1 kg for small fruits (total of 60 fruits) [25,26]. In the first portion, the commercial quality of the fruit was evaluated, taking into account the weight and size (equatorial and longitudinal diameter) of the whole fruit. In addition, pH, soluble solids, total titratable acid, moisture, and ash were measured in the pulp.

For the second portion, the edible part of the fruits was removed, cut into small pieces, and placed in plastic tubes to be frozen at −80 °C before freeze-drying using a Christ Alpha 1-4 LDplus (GmbH, Osterode am Harz, Germany). The freeze-dried sample was hermetically sealed in amber glass jars under a nitrogen atmosphere and stored at −21 °C until analysed for bioactive compounds and antioxidant activity [2].

In whole fruit, fruit weight was accurately recorded using a Mettler Toledo ML204T/00 balance (Mettler Toledo, Greifensee, Switzerland), while the size was assessed using a Titan 23175 digital calliper (Titan, Kent, WA, USA) [27]. On the other hand, the edible part of the fruit was crushed in a mortar and pestle. The liquid portion was measured for pH using a SevenMultiTM S47 pH meter (Mettler Toledo, Greifensee, Switzerland) [28], and the soluble solids were determined using a handheld refractometer (Boeco, Germany). In the whole pulp, total titratable acid was determined by titration [29], while moisture and ash were determined by gravimetry using a Be20 oven with air circulation (Memmert GmbH Co.KG, Schwabach, Germany) at 70 °C and a Thermolvne muffle (Thermo Fisher Scientific, Waltham, MA, USA) at 550 °C [2].

### 2.3. Analysis of Bioactive Compounds

#### 2.3.1. Vitamin C

Quantifying vitamin C followed the NSAI protocol with some modifications [30] and was performed in triplicate. A 100 mg of lyophilised powder was weighed and mixed with 0.2 mL of 0.2% *DL*-homocysteine and 1.2 mL of 3% metaphosphoric acid. The resulting mixture was vortexed and shaken in a Fisher Scientific FS60 ultrastat (Fisher Scientific, Hampton, NH, USA) for 7 min. The mixture was then made up to 2 mL with deionised water. The solids were separated by centrifugation at 13,171× *g* in a MiniSpin series microcentrifuge (Eppendorf, Hamburg, Germany) conditioned at 4 °C for 5 min. The supernatant was filtered through a 0.45 µm PVDF (polyvinylidene fluoride) filter and transferred to a 2 mL vial for quantification on an Agilent 1200 series rapid resolution liquid chromatograph (RRLC) (Agilent Technologies, Santa Clara, CA, USA) coupled to a DAD-UV-Vis detector at 244 nm. Vitamin separation was performed on a Zorbax Eclipse column, XDB-C18, 80 Å (4.6 × 50 mm, 1.8 µm, 600 bar) (Agilent Technologies, Santa Clara, CA, USA), conditioned at 30 °C and with a phase flow rate of 1 mL/min. The mobile phase consisted of a 90:10 solution of 1.5% monobasic potassium phosphate monobasic and 1.8% n-acetyl-*n*,*n*,*n*-trimethylammonium bromide. Injecting 20 µL of sample, and the run time was 20 min. Injections were duplicated, and chromatograms were monitored using Open Lab ChemStation software. Vitamin C was identified by comparison of retention time, spectra at 244 nm, and internal standard. A calibration curve was used with a 1 mg/mL solution of *L*-(+)-ascorbic acid standard, constructed with different injection volumes (3, 5, 10, 15, and 20 µL) for quantification. Vitamin C was expressed as milligrams per 100 g of dry matter (mg/100 g DM) [2].

#### 2.3.2. Organic Acid

The quantification of organic acids was carried out according to the Macrae protocol with some modifications [31]. In triplicate, 40 mg of lyophilised powder was weighed and mixed with 1.5 mL of 0.02 N sulphuric acid containing 0.05% metaphosphoric acid and 0.02% *DL*-homocysteine. The mixture was vortexed and then sonicated for 3 min. The mixture was then made up to 2 mL with deionised water. The solids were separated by centrifugation at 13,171× *g* at 4 °C for 5 min. The supernatant was filtered through a 0.45 µm PVDF filter and transferred to a 2 mL vial for quantification on an Agilent 1200 series RRLC coupled to a DAD-UV-Vis detector at 210 nm. Separation of the organic acids was performed on a YMC-Triart C18 column (150 × 4.6 mm, 3, 12 nm, 400 bar) (YMC Europe GmbH, Dinslaken, Germany) conditioned at 30 °C with a phase flow rate of 1 mL/min. The mobile phase was a 0.027% sulphuric acid solution. Injecting 20 µL of sample, and the run time was 30 min. The injection was performed in duplicate, and the chromatograms were monitored using the Open Lab ChemStation software. Organic acids were identified by comparison of retention time, spectrum at 210 nm, and internal standard. For quantification, a calibration curve was used with a 100 mg/mL solution of citric acid, malic acid, and *L*-(+)-tartaric acid standards, prepared separately with different injection volumes (3, 5, 10, 15, and 20 µL). Each organic acid was expressed in grams per 100 g of dry weight (g/100 g DW). Total organic acids were calculated by summing the concentrations of the individual compounds.

#### 2.3.3. Carotenoids

The carotenoids were quantified according to the protocol of Coyago [2] with some modifications. A 3 × 2 experimental design was used to select the extraction solvent, considering different solvents (hexane: acetone (1:1), methanol: acetone: dichloromethane (1:1:2) and methanol: trichloromethane: water (1:2:1)) and ultrasonic extraction times (1, 2 and 3 min). For extraction, 20 mg of lyophilised powder was weighed in triplicate and mixed with the selected solvent. The mixture was vortexed and then sonicated for 2 min. The solids were separated by centrifugation at 13,171× *g* at 4 °C for 5 min. The supernatant was collected, and the solid was subjected to several extractions until the colour was removed entirely. The supernatant was then evaporated to dryness at a temperature not exceeding 30 °C under vacuum using a Buchi TM R-100 (Fisher Scientific, Hampton, NH, USA). The dried extract was redissolved with 40 µL of ethyl acetate for quantification on an Agilent 1200 series RRLC coupled to a DAD-UV-Vis detector. The carotenoid separation was performed on a C18 Poroshell 120 column (2.7 µm, 5 cm × 4.6 mm) (Agilent Technologies, Santa Clara, CA, USA) conditioned at 30 °C with a 1 mL/min phase flow rate. The mobile phase consisted of a mixture of acetonitrile (solvent A), methanol (solvent B) and ethyl acetate (solvent C) with a linear gradient of 85% A + 15% B at 0 min; 60% A + 20% B + 20% C at 5 min; 60% A + 20% B + 20% C at 7 min; 85% A + 15% B at 9 min; 85% A + 15% B at 12 min. In duplicate, 10 µL of sample was injected and chromatograms were monitored using Open lab ChemStation software (Version 2.15.26). The carotenoids were identified by comparison of the retention times of the respective spectra at 350 nm or 450 nm. For quantification, a calibration curve was used with a 1 mg/mL standard solution of β-carotene, β-cryptoxanthin, lutein, lycopene, and zeaxanthin, separately constructed with different injection volumes (3, 5, 10, 15, and 20 µL). Each carotenoid was expressed in milligrams per 100 g of dry weight (mg/100 g DW). Total carotenoids were calculated by summing the individual concentrations of the identified compounds.

#### 2.3.4. Phenolics

The quantification of phenolics was carried out according to the protocol of Coyago [2] with some modifications. A 3 × 2 design was used to select the extraction solvent, considering different solvents (100% methanol, acetone-ethanol (1:1), and methanol acidified with 0.1% HCl) and ultrasonic extraction times (1, 3, and 5 min). In triplicate, 40 mg of lyophilised powder was weighed and mixed with 1.0 mL of the selected selection. The mixture was vortexed and then sonicated for 3 min. The solids were separated by centrifugation at 13,171× *g* at 4 °C for 5 min. The supernatant was collected, and the solid was subjected to two further extractions with 500 µL of the selected solution. The supernatant was then filtered through a 0.45 µm PVDF filter and transferred to a 2 mL vial for quantification on an Agilent 1200 series RRLC coupled to a DAD-UV-Vis detector with a wavelength scan between 220 and 500 nm. The separation of phenolic compounds was performed on a Zorbax Eclipse Plus C18 column (4.6 × 150 mm, 5 µm) (Agilent Technologies, Santa Clara, CA, USA) conditioned at 30 °C and with a phase flow rate of 1 mL/min. The mobile phase consisted of a 0.01% aqueous solution of 0.01% aqueous solution of formic acid (solvent A) and acetonitrile (solvent B) using a linear gradient of 100% at 0 min; 95% A + 5% B at 5 min; 50% A + 50% B at 20 min; washing and rebalancing the column at 30 min. In duplicate, 10 µL of sample was injected and chromatograms were monitored using Open Lab ChemStation software. Phenolic compounds were identified by comparison of retention time corresponding spectra at 280 nm, 320, or 370 nm, as appropriate. For quantification, a calibration curve was used with a 1 mg/mL solution of caffeic acid, chlorogenic acid, chrysin, *p*-coumaric acid, m-coumaric acid, o-coumaric acid, ferulic acid, gallic acid, *p*-hydroxybenzoic acid, 3-hydroxybenzoic acid, 2-methoxybenzoic acid, 3-methoxybenzoic acid, 3-hydroxybenzoic acid, 2,5-dihydroxybenzoic acid, kaempferol, luteolin, naringin, quercetin, rutin, shikimic acid, syringic acid, and vanillic acid, separately prepared with different injection volumes (3, 5, 10, 15, and 20 µL). Each phenolic compound was expressed in milligrams per gram of dry weight (mg/g DW). Total phenolics were calculated as the sum of the individual concentrations of the identified compounds.

### 2.4. Antioxidant Activity Analyses

Using the ABTS method, 100 mg of lyophilised powder was weighed and mixed with 0.8 mL of 50% aqueous methanol solution to quantify antioxidant activity. The mixture was vortexed and then sonicated for 2 min. The solids were separated by centrifugation at 13,171× *g* at 4 °C for 3 min. The supernatant was collected, and the solid was subjected to a second extraction with 0.8 mL of a 56% aqueous acetone solution. The resulting supernatant was filtered through a 0.45 µm PVDF filter and stored under refrigeration until analysis. For the generation of the ABTS^+^ radical, a 1:1 solution of 7 mM ABTS and 2.45 mM potassium persulfate was prepared and kept in the dark for 16 h. The ABTS^+^ radical was then diluted in a 1/10 ratio with absolute ethanol until the absorbance reached a value of 0.7 at 734 nm [32,33]. The calibration curve was constructed using a stock solution of 2.5 nM Trolox standard dissolved in ethanol, and dilutions of 0, 12.5, 25, 50, and 75 µL were made in 300 µL. Finally, 20 µL of the sample and 280 µL of the ABTS^+^ radical solution were added to a 96-well VWT microplate (Corning, Glendale, AZ, USA) [34]. Absorbance was measured at 734 nm using a Thermo Scientific Multiskan GO (Agilent Scientific Instruments, Santa Clara, CA, USA). microplate reader spectrophotometer Antioxidant activity was expressed as millimolar Trolox equivalents per gram of dry weight (µmol TE/g DW).

On the other hand, the quantification of antioxidant activity by the DPPH method followed the protocol of Pires with some modifications [35]. Twenty mg of lyophilised powder was weighed and mixed with 2 mL of methanol. The mixture was vortexed and then sonicated for 3 min. The supernatant was separated by centrifugation at 13,171× *g* at 4 °C for 3 min. It was then filtered through a 0.45 µm PVDF filter and stored refrigerated until analysis. The calibration curve was constructed using a stock solution of 1 mg/mL *L*-(+)-ascorbic acid standard dissolved in methanol and diluted to 50%. The stock solution was diluted in the range of 0 to 6 mg/mL. To generate the DPPH radical, 10 mg of DPPH was weighed and dissolved in 50 mL of methanol. Finally, 20 µL of the sample and 280 µL of the DPPH radical solution were added to a 96-well VWT microplate for both standards and samples. Absorbance was measured after 30 min of shaking on a Shaker 4310 orbital shaker (Fisher Scientific, Waltham, MA, USA) in the dark at 560 nm using a BioTek H1 spectrophotometer (Agilent Scientific Instruments, CA, USA). Antioxidant activity was expressed as millimolar ascorbic acid equivalents per gram of dry weight (µmol AAE/g DW).

### 2.5. Statistical Analysis

Statistical analysis was performed using STATGRAPHICS Centurion XVII, SIGMAPLOT 14.0, and RStudio (version 4.2.3). Results were expressed as mean ± standard deviation. Tukey’s test with a significance level of 0.01 was used to determine significant differences between means. A 3 × 2 factorial design was used for solvent selection, and response surface methodology was used to optimise the response using continuous (time) and categorical (solvent type) factors. In addition, Pearson correlation was performed at the 99% confidence level to identify possible associations between variables. Principal component analysis (PCA) was also used to determine the most influential variables in the study.

## 3. Results

### 3.1. Physico-Chemical Analyses

Information on the commercial quality parameters of the fifty-one fruits studied is presented in Table 2, covering aspects such as fruit weight, equatorial and longitudinal diameters, pH, soluble solids content, total titratable acid content, moisture content, ash content, and colour intensity, both in the external and internal parts of the fruit.

#### 3.1.1. Weight

The weight of the fruits in this study (Table 2) varied considerably, from lighter tropical fruits such as kulkas (*M. crocea*) weighing 0.4 g, to considerably heavier fruits, such as jackfruit (*A. heterophyllus*) weighing 9117.2 g. On the other hand, fruits such as calabaza (*S. odorifera*), smooth-skinned cacao (*T. cacao* yellow), badea (*P. quadrangularis*), and jackfruit (*A. heterophyllus*) had significantly high weights, suggesting considerable nutrient and water storage capacity. In contrast, some fruits, such as wild blackberry (*R. niveus*), raspberry (*R. rosifolius*), and kulkas (*M. crocea*), had remarkably low weights. Despite their small size, these fruits may have a high concentration of bioactive compounds, as previous research has shown that small tomatoes (*S. lycopersicum*) accumulate large amounts of bioactive compounds in their structures compared to larger tomatoes [19]. The results of this study are consistent with previous research. For example, weights similar to those reported by other authors were observed for sapote (*Q. cordata*) (373 g to 1088 g) [36], jackfruit (2.0 to 20 kg) [37], yellow tree tomato (between 75.9 and 160.4 g) and purple tree tomato (between 105.7 and 144.4 g) [2], and ripe cereza agria (*D. hebecarpa*) (4.5 g in July and 6.9 g in April) [38]. Tree blackberry (*M. alba*) ranged from 1.3 to 3.7 g [39], while pitahaya (*S. megalanthus*) ranged from 277.2 to 556.8 g [40]. It should be noted that fruit weight is influenced by factors such as variety, maturity agronomic, and environmental conditions [2,41,42].

#### 3.1.2. Size

The tropical fruits studied presented a diversity of sizes (Table 2), ranging from small fruits, such as kulkas and wild blackberries (*R. niveus*) with a longitudinal diameter of 1.0 cm, to considerably larger fruits, such as jackfruit (*I. edulis*) with a longitudinal diameter of 88.4 cm and an equatorial diameter of 27.5 cm in the case of jackfruit (*A. heterophyllus*).

Fruits such as badea (*P. quadrangularis*), calabaza (*S. odorifera*), jackfruit (*A. heterophyllus*) and guaba de bejuco (*I. edulis*) had remarkably large longitudinal diameters. Fruits with smaller longitudinal diameters were identified, such as cereza agria (*D. hebecarpa*), uvilla (*P. peruviana*), raspberry (*R. rosifolius*), capuli (*P. salicifolia*), wild blackberry (*R. niveus*) and kulkas (*M. crocea*). In addition, the results of this study are consistent with previous research. For example, similarities were observed in the equatorial (4.7 to 6.9 cm) and longitudinal (5.8 to 7.7 cm) diameters of yellow tree tomatoes. In comparison, purple tree tomatoes ranged from 5.3 cm to 6.0 cm and 7.1 cm to 7.9 cm, respectively [2]. For sapote (*Q. cordata*), dimensions ranged from 10.1 to 11.1 cm longitudinal diameter and 8.1 to 10.7 cm equatorial diameter [43]. For sour cherry, an equatorial diameter of 2.1 cm in April and 1.9 cm in July was recorded, with a longitudinal diameter of 2.4 cm in April and 2.1 cm in July [38]. On the other hand, bramble (*M. alba*) had an equatorial diameter of 1.6 to 3.0 cm and a longitudinal diameter of 0.8 to 1.6 cm [39]. It is important to consider that agronomic, geographical, environmental conditions, and maturity stage conditions influence fruit size, resulting in different dimensions for the same species [27,44].

#### 3.1.3. pH

Table 2 presents the pH values of the tropical fruits under study, revealing a remarkable variability of this parameter. Values ranged from a pH of 1.0 in wild blackberry (*R. niveus*) to 8.2 in white chayote (*S. edulis*). The pH of orange chonta (*B. gasipaes*), calabaza (*S. odorifera*), ungurahua (*O. bataua*), sapote (*M. cordata*) and white chayote (*S. edulis*) was aboveneutral. Fruits such as membrillo (*C. oblonga*), yellow taxo (*P. tripartita*), yellow ovo (*S. purpurea* yellow), cereza agria (*D. hebecarpa*), tree mulberry (*M. alba*) and wild blackberry (*R. niveus*) had pH values below 3, suggesting that these fruits may be less susceptible to attack by microorganisms [45]. However, fruits with milder flavours had pH values closer to neutral. The results of this study are related to previous research. For example, similarities were observed in the pH of yellow tree tomatoes, which ranged from 3.2 to 6.0, and purple tree tomatoes, which ranged from 3.4 to 4.4 [2]. For sapote (*Q. cordata*), pH values ranging from 6.5 to 7.0 were found [43]. Other fruits, such as granadilla de monte (*P. edulis*), had a pH of 2.9 [46], while chamburo (*V. pubescens*) had a pH of 4.1 [47]. In the case of pitahaya (*S. megalanthus*), a range of 15.3 to 20.7 °Brix was recorded [40].

#### 3.1.4. Soluble Solid

The soluble solids of the tropical fruits in this study ranged from 2.2 °Brix in ungurahua (*O. bataua*) to 28.6 °Brix in mamey (*P. sapota*) (Table 2). Thus, fruits with highly soluble solids, such as rough skin cacao (*H. nitida*), yellow caimito (*P. caimito*), granadilla de monte (*P. edulis*), and mamey (*P. sapota*), had a sweeter taste. Fruits with more soluble solids tend to be more attractive to consumers because of their pronounced sweetness. In contrast, ungurahua (*O. bataua*), copal (*D. peruviana*), white chayote (*S. edulis*), and raspberry (*R. rosifolius*), which had lower soluble solids, did not have a sweet taste.

In this sense, the soluble solids of the tropical fruits in this study provide valuable information on the profile of water-soluble compounds, mainly sugars, which influence the flavour, the sensory quality of the fruit, and the degree of ripeness of the fruit, so that ripe fruits have a higher concentration of soluble solids than those that are not full ripe [48].

The results of this study are also in line with previous research. For example, similarities were observed in the soluble solids of yellow tree tomatoes, which ranged from 8.2 to 12.5 °Brix, and vine tomatoes, which ranged from 8.7 to 11.1 °Brix [2]. For sour cherry, 12.6 °Brix was recorded in ripe fruits [38]. Red ovo (*S. purpurea*) had a soluble solids content of 21.6 °Brix [49]. Granadilla de monte (*P. edulis*) had a value of 13.5 °Brix [46], while chamburo (*V. pubescens*) had a value of 5.0 °Brix [47].

#### 3.1.5. Total Titratable Acid

The titratable acidity of tropical fruits ranged from 0.1% in fruits such as red ovo (*S. purpurea* red), white chayote (*S. edulis*), guaba de bejuco (*I. edulis*) and guaba (*I. insignis*) to 4.6% in arazá (*E. stipitata*) (Table 2). Titratable acidity is a parameter that indicates the number of organic acids that determine the flavour and organoleptic characteristics of fruit [45]. In this respect, high percentages of titratable acidity were found in fruits such as chicle (*L. lactescens*), sour cereza agria (*D. hebecarpa*), cocona (*S. sessiliflorum*) and arazá (*E. stipitata*). In contrast, several fruits showed low titratable acidity values, suggesting a less acidic and possibly sweeter flavour profile than others. Furthermore, the results of this study were consistent with previous research. For example, similarities in the percentage of total titratable acidity were observed in yellow tree tomatoes, ranging from 0.8 to 1.9%, and purple tree tomatoes, ranging from 0.6 to 1.3% [2]. In sapote (*Q. cordata*), values ranging from 0.09 to 0.18% were found [43]. Red ovo (*S. purpurea*) showed a titratable % acidity of 9.9% [49]. Granadilla de monte (*P. edulis*) had a value of 3.2% [46], while chamburo (*V. pubescens*) had a value of 0.1% [47].

#### 3.1.6. Humidity

Moisture in the tropical fruits studied ranged from a minimum of 37.8% in white chayote (*S. edulis*) to a maximum of 96.5% in red ovo (*S. purpurea* red) (Table 2). Moisture plays a critical role in the quality and shelf-life of fruit, directly affecting aspects such as texture, and freshness [50]. Fruits such as chamburo (*V. pubescens*), momórdica (*M. charantia*), granadilla de monte (*P. edulis*) and ovo rojo (*S. purpurea* red) have high moisture percentages, indicating a significant water content. This gives them a juicy and fresh texture but makes them more susceptible to spoilage if not appropriately handled. On the other hand, fruits such as white chayote (*S. edulis*), red cacao (*T. cacao* red), red pomarrosa (*S. jambos* red), yellow caimito (*P. caimito*), smooth skin cacao (*T. cacao* yellow) and chicle (*L. lactescens*) have relatively low moisture percentages, indicating a lower water content and therefore a denser or firmer texture and possibly less attack by micro-organisms, thereby increasing the shelf-life of the fruit. [50]. In addition, the results of this study are consistent with previous research. For example, similarities were observed in the moisture content of yellow tree tomatoes, which ranged from 79.2% to 98.3%, and purple tree tomatoes, which ranged from 77.5% to 87.8% [2]. Sapote (*Q. cordata*) pulp had a moisture content of 85.0% [43]. Sour cherry had a moisture content of 87.1% in April and 84.4% in July [38]. Red ovo (*S. purpurea*) had a moisture content of 70.9% [49]. In purple taxo (*P. mollissima*), values ranging from 77.9% to 93.0% have been reported by several authors [51]. Uvilla (*P. peruviana*) showed a moisture range from 82.4% to 85.9% [52]. Passion fruit (*P. edulis*) had a moisture content of 81.7% [46]. Mulberry (*M. alba*) ranged from 82.5% to 86.5% [39]. Chamburo (*V. pubescens*) had a moisture content of 91.6% [47]. Finally, pitajaya (*S. megalanthus*) showed a moisture variability between 82.3% and 89.0% [40].

#### 3.1.7. Ash

Ash is an important indicator of the fruit’s presence of minerals and other inorganic compounds [53]. Thus, Table 2 shows that this percentage ranged from 0.3% in arazá (*E. stipitata*) and achotillo (*N. lappaceum*) to 20.1% in red cacao (*T. cacao* red). Smooth skin cacao (*T. cacao* yellow), copal (*D. peruviana*), and red cacao (*T. cacao* red) thus showed high ash concentrations, possibly influenced by the type of fruit, the growing conditions, and the composition of the soil in which they were grown, as tropical fruits have been shown to be an important source of minerals [4]. On the other hand, it is important to note that the results of this study were consistent with previous research. For example, similarities in ash content were observed for yellow tree tomatoes, ranging from 1.3% to 13.2%, and purple tree tomatoes, ranging from 0.9% to 3.4% [2]. Sapote (*Q. cordata*) pulp had an ash content of 0.8% [37]. Sour cherry had an ash content of 0.5% in April and 0.4% in July [38]. Red ovo (*S. purpurea*) had an ash content of 2.3% [49]. In purple taxo (*P. mollissima*), values between 0.3% and 0.7% have been reported by several authors [51]. In uvilla (*P. peruviana*), the ash ranged from 0.7% to 0.8% [52]. Black passion fruit (*P. edulis*) showed a value of 0.6% ash [46]. Tree blackberry (*M. alba*) varied between 3.6% and 7.1% [39]. Finally, pitahaya (*S. megalanthus*) showed an ash range of 0.4% to 0.6% [40]. In this sense, it is important to stress that the ash content can vary according to the agronomic conditions of the crop, the supply of minerals during plant growth, and the characteristics of the soil, which influences the amount of minerals supplied, as shown by other studies [2]. Therefore, the variability in ash levels between different fruits suggests that the nature of the fruit and the growing environment may influence these levels.

### 3.2. Analysis of Bioactive Compounds

#### 3.2.1. Vitamin C

Vitamin C, also known as ascorbic acid, is an abundant vitamin in fruits and is essential for human health due to its essential role as an antioxidant, contributing to immune function and metabolism [14]. The concentration of vitamin C in the fruits studied showed a wide range of values, from the undetectable limit to 768.2 mg/100 g dry weight (DW) in sour cherry (*D. hebecarpa*) (Table 3). On the other hand, some tropical fruits, such as yellow guava (*P. guineense*), red guava (*P. guajava*), and cereza agria (*D. hebecarpa*), have significantly high vitamin C concentrations, exceeding 400 mg/100 g DW. The high concentrations are particularly interesting as they more than meet the adult daily requirement for vitamin C, which is 90 mg vitamin C per 100 g ration for men and 75 mg vitamin C per 100 g ration for women [51]. The significant presence of vitamin C in these tropical fruits underlines their importance in the diet, especially in regions where these fruits are regularly consumed. The results of this study correlate with previous research. For example, similar levels of vitamin C were found in yellow tree tomatoes, ranging from 34.7 to 217.6 mg/100 g dry weight, and in purple tree tomatoes, ranging from 56.7 to 175.0 mg/100 g dry weight [2]. A range of 10.2 to 12.5 mg/100 g dry weight was found in sapote (*Q. cordata*) [43]. Similarly, a range of 24 to 75 mg/100 g dry weight was found in cereza agria (*D. hebecarpa*). Uvilla (*P. peruviana*) showed a value of 20 mg/100 g DW [52]. Granadilla de monte (*P. edulis*) showed a value of 25.5 mg/100 g DW [20]. Finally, pitahaya (*S. megalanthus*) ranged from 7.3 to 25.8 mg/100 g DW [40].

#### 3.2.2. Organic Acid

The concentration of organic acids in tropical fruits in this study ranged from 2.3 g/100 g DW in copal (*D. peruviana*) to 37.2 g/100 g DW in yellow taxo (*P. tripartita*) (Table 3). Fruits such as salak (*S. zalacca*), black moriche (*M. flexuosa*), arazá (*E. stipitata*), and yellow taxo (*P. tripartita*) showed concentrations higher than 25.0 g/100 g DW. At the same time, the results shown in this study were related to other studies; for example, the concentration of total organic acids in yellow tree tomato ranged from 48.4 to 94.0 g/100 g DW, while in purple tree tomato, it ranged from 42.0 to 115.8 g/100 g DW [2]. Notably, the concentration of organic acids may vary with the degree of fruit ripeness, as they accumulate during phenolic development but decrease during storage [54]. In addition, higher concentrations of organic acids are associated with low concentrations of soluble solids, as organic acids are produced from sugars during photosynthesis; therefore, the lower the soluble solids, the higher the total organic compounds [55]. Tropical fruits are, therefore, an important source of organic acids, which in some cases give them their characteristic sour taste and contribute to the health of consumers, as organic acids have been shown to have antioxidant, antibacterial, and anti-inflammatory properties [20].

On the other hand, tree blackberry (*M. alba*), cocona (*S. sessiliflorum*), granadilla de monte (*P. edulis*), and yellow taxo (*P. tripartita*) showed particularly high concentrations of citric acid, exceeding 10 g/100 g DW. These levels indicate that these tropical fruits have a pronounced sour taste, which may contribute to their characteristic flavour profile, preservative properties, antimicrobial and consumer appeal, and health-promoting properties [56].

Malic acid concentrations ranged from 0.5 g/100 g dry weight in tuna (*O. ficus-indica*), guaba de bejuco (*I. edulis*), mamey (*P. sapota*), purple and yellow tree tomatoes (*S. betaceum*) to 25.0 g/100 g dry weight in arazá (*E. stipitata*). Fruits such as sour cereza agria (*D. hebecarpa*), salak (*S. zalacca*), brown moriche (*M. flexuosa*), and arazá (*E. stipitata*) showed remarkably high concentrations of malic acid, exceeding 15 g/100 g DW.

Tartaric acid concentrations ranged from 0.1 g/100 g dry weight in persimmon (*D. kaki*) to 9.9 g/100 g dry weight in red pomarrosa (*S. jambos* red). Tartaric acid is an important organic acid that contributes to the acidic taste of many fruits and is critical to the organoleptic quality of these foods. Although this acid is rare in plant species, it is found in high concentrations in sweet cherry, tamarind, blueberry, and some citrus fruits, giving the fruit special characteristics. Although the pathway of tartaric acid accumulation is not fully understood, it is believed that one of the metabolic pathways involved is that of ascorbic acid [57,58]. This study shows that some fruits, such as purple and yellow tree tomato (*S. betaceum*), uva de chonta (*B. concinna*), and red pomarrosa (*S. jambos* red), have significantly high concentrations, exceeding 7.0 g/100 g DW.

#### 3.2.3. Carotenoids

**Figure 1:**
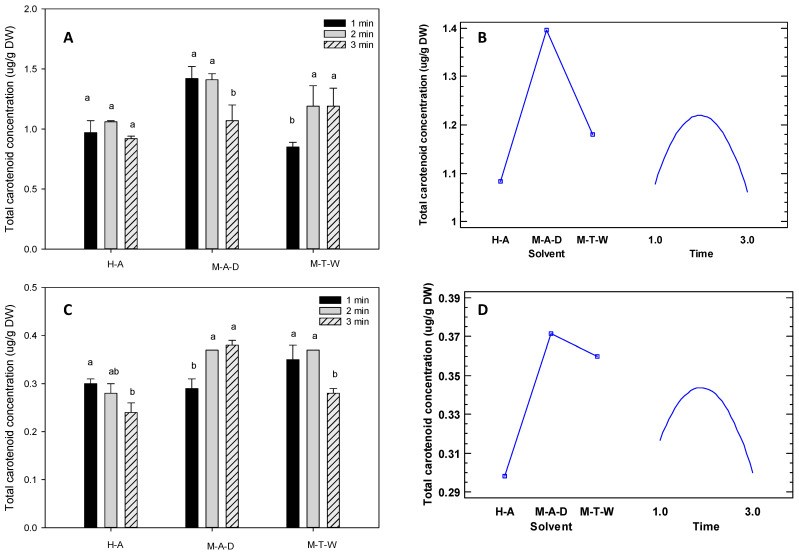
Results of the experimental design for the extraction of carotenoids. Note: Figures (**A**,**B**) *Matisia cordata*; Figures (**C**,**D**), *Spondias purpurea*; H-A, n-hexane: acetone (1:1); M-A-D, methanol: acetone: dichloromethane (1:1:2); M-T-W, methanol, trichloromethane, water (1:2:1). The lower case letters next to the standard deviation indicate the separation of the mean values of the tropical fruits studied at a 95% confidence level.Time in minutes.

Carotenoids are bioactive compounds in many tropical fruits responsible for their different colours, from yellow and orange to red and purple. In addition, some carotenoids are provitamins and have antioxidant activity [27,44]. Therefore, Figure 1 show the experimental design results for extracting carotenoids from *M. cordata* and *S. purpurea*. The results indicated that the solvent was the most influential factor in the extraction of carotenoids, followed by a solvent-time interaction. These results were similar to those found in other studies, which concluded that solvent, extraction temperature, and time influence the final concentration of carotenoids extracted form a given matrix [59,60]. In this regard, the experimental design showed that a solvent combination of methanol: acetone: dichloromethane (1:1:2), together with an extraction time of 2 min, produced the highest concentration of carotenoids.

On the other hand, Table 4 shows the specific profile of carotenoids in each of the fruits studied. Thus, the concentration of carotenoids, as the sum of the individual compounds, ranged non-detectable limits in fruits such as ungurahua (*O. batata*) and copal (*D. peruviana*), which are characterised by opaque colours (dark purple) to fruits with high concentrations such as momórdica (*M. charantia*) and calabaza (*S. odorifera*), which have an orange colour.

When certain fruits were compared with the rest of the fruits studied, significant concentrations of individual carotenoids were observed. For example, chicle (*L. lactescens*) was characterised by a high concentration of α-carotene (284.0 mg/100 g DW), purple taxo (*P. mollissima*) presented a high concentration of β-carotene (86.0 mg/100 g DW), mamey had a high concentration of β-cryptoxanthin (43.0 mg/100 g DW), orange chonta had a high concentration of luteoxanthin (2.0 mg/100 g DW), momórdica (*M. charantia*) had a high concentration of lutein (16.0 mg/100 g DW) and lycopene (465.0 mg/100 g DW), calabaza (*S. odorifera*) had a high concentration of phytoene (311.0 mg/100 g DW), yellow ovo (*S. purpurea* yellow) had a high concentration of violaxanthin (3.0 mg/100 g DW), uvilla (*P. peruviana*) had a high concentration of zeaxanthin (3.0 mg/100 g DW), and uva de monte (*P. cecropiifolia*) had a high concentration of zeinoxanthin (91.0 mg/100 g DW). Several studies support the significant presence of β-carotene, lycopene, lutein, and zeaxanthin in these fruits [42,51,61,62].

Furthermore, the results obtained in this study are consistent with other studies. For example, the concentration of total carotenoids in yellow tree tomatoes ranged from 44.2 to 390.7 mg/100 g DW, while in purple tree tomatoes, it ranged from 67.4 to 208.7 mg/100 g DW [2]. Total carotenoid concentrations ranging from 0.7 to 1.3 µg total carotenoids/g were also found in sapote (*Q. cordata*) [43], and purple taxo (*P. mollissima*) showed a concentration of 118.8 mg β-carotene/100 g DW [51].

#### 3.2.4. Phenolics

**Figure 2:**
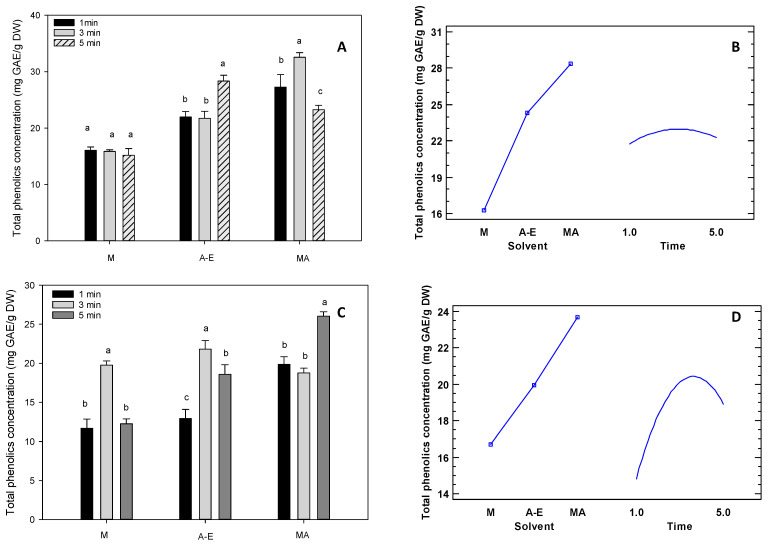
Results of the experimental design for the extraction of phenolics. Note: Figures (**A**,**B**), *Dacryodes peruviana*; Figures (**C**,**D**), *Syzygium jambos*; M, 100% methanol; A-E, acetone: ethanol (1:1); MA, 80% acidified methanol with 0.1% HCl. The lower case letters next to the standard deviation indicate the separation of the mean values of the tropical fruits studied at a 95% confidence level. Time in minutes.

Phenolic compounds are an important category of bioactive compounds found in tropical fruits, they provide colour and have antioxidant properties and are, therefore, considered beneficial to human health [27,63]. Figure 2 shows the experimental design results for extracting phenolic compounds from *D. peruviana* and *S. jambos*. The results indicated that the solvent was the most influential factor in the extraction of phenolic compounds, followed by a solvent-time interaction. In this regard, the experimental design showed that the solvent that produced the highest concentration of total phenolics was an 80% methanol solution acidified with 0.1% hydrochloric acid, together with an extraction time of 3 min.

On the other hand, Table 5 details the specific profile of phenolic compounds of the tropical fruits studied. The concentration of total phenolic compounds, as the sum of the individual compounds, ranged from 0.3 mg/g DW in jackfruit (*A. heterophyllus*) to 535.4 mg/g DW in achotillo (*N. lappaceum*). Other fruits such as white chayote (*S. edulis*) (487.0 mg/g DW) and granadilla de monte (*P. edulis*) (114.0 mg/g DW) also showed high concentrations of total phenolics. These results confirm the findings of other studies that phenolic compounds are the most abundant in tropical fruits [15].

When certain fruits were compared with the rest of the fruits studied, significant concentrations of individual phenolic compounds were observed. For example, achotillo (*N. lappaceum*) was characterised by high concentrations of chlorogenic acid (169.0 mg/g DW) and ferulic acid (366.5 mg/g DW). Chicle (*L. lactescens*) had a high concentration of naringenin (4.0 mg/g DW), while white chayote (*S. edulis*) had a high concentration of quercetin (91.1 mg/g DW) and rutin (237.3 mg/g DW). Similarly, rough-skinned cacao (*H. nitida*) showed a high concentration of syringic acid (10.3 mg/g DW), openers a high concentration of caffeic acid (30.7 mg/g DW), arazá (*E. stipitata*) a high concentration of m-coumaric acid (17.9 mg/g DW), granadilla de monte (*P. edulis*) had a high concentration of *p*-coumaric acid (0.6 mg/g DW), cereza agria (*D. hebecarpa*) had a high concentration of *p*-hydroxybenzoic acid (27.3 mg/g DW), and red pomarrosa (*S. jambos* red) had a high concentration of gallic acid (9.0 mg/g DW). It is important to note that high concentrations of ferulic acid may be related to the biosynthesis of phenolic compounds, indicating a metabolic pathway in which caffeic acid is previously formed, leading to the formation of ferulic acid and contributing to a higher concentration of this compound [44].

Furthermore, the results of this study agree with previous studies. For example, the concentration of total phenolic compounds in yellow tree tomato ranged from 13.08 to 235.8 mg/100 g dry weight (DW), while in purple tree tomato, it ranged from 30.8 to 133.2 mg/100 g DW [2]. Concentrations of total phenolic compounds were also found to range from 8.5 to 11.1 mg gallic acid equivalent (GAE)/100 g of sapote (*Q. cordata*) pulp [43]. Tart cherry showed a total phenolic content of 195.0 mg GAE/100 g fresh fruit (FW) in April and 239.0 mg GAE/100 g in July [38]. On the other hand, purple taxus (*P. mollissima*) showed a concentration range from 460.1 mg GAE/100 g FW to 5012.8 mg GAE/100 g FW [51], and chamburo (*V. pubescens*) showed a range from 23.8 to 129.1 mg GAE/100 g [47].

It is important to note that high ripening indices are associated with a higher production of phenolic compounds, as sugars act as a substrate for anthocyanin synthesis, and acids stimulate the synthesis potential, which is essential for fruit colouring [64].

### 3.3. Antioxidant Activity Analyses

The data concerning the antioxidant activity of the fifty-one fruits studied, obtained using the ABTS and DPPH methods, are summarised in Table 3. The antioxidant activity, measured by the ABTS method, of the fruits studied varied widely, ranging from 3.2 µmol Trolox equivalents (TE)/g dry weight (DW) in uvilla (*P. peruviana*) to 161.4 µmol TE/g DW in uva de monte (*P. cecropiifolia*). The antioxidant activity measured by the DPPH method also showed remarkable variations in the fruits studied, ranging from 0.8 µmol AAE/g DW in maracuyá-badea (*P. hybrid*) to 80.5 µmol AAE/g DW in tree blackberry (*M. alba*). Interestingly, fruits such as tree mulberry (*M. alba*), tree tomato (*S. betaceum*), black moriche (*M. flexuosa*), and uva de monte (*P. cecropiifolia*) showed a concentration higher than 115.8 µmol TE/g DW when the ABTS method was used (Table 3). This parameter is essential as it indicates the ability of tropical fruits to neutralise free radicals and protect cells from oxidative damage.

On the other hand, some fruits, such as black moriche (*M. flexuosa*), wild blackberry (*R. niveus*), purple taxo (*P. mollissima*), and tree blackberry (*M. alba*), showed concentrations higher than 38.8 µmol AAE/g DW when the DPPH method was used. In this sense, the presence of antioxidants in fruits, such as vitamins, polyphenols, flavonoids, and carotenoids, contributes to their health-promoting properties. Regular consumption of fruits with high antioxidant activity can be beneficial in reducing oxidative stress in the body, which is associated with various chronic diseases and ageing. In this sense, these tropical fruits are becoming valuable functional foods that can contribute to a balanced diet and disease prevention [20].

Furthermore, the results obtained in this study are consistent with previous research. For example, the antioxidant activity measured by the ABTS method in yellow tree tomatoes ranged from 4.4 to 119.7 µmol Trolox equivalents (TE)/g DW. In contrast, it ranged from 3.4 to 137.9 µmol TE/g DW in violet tree tomatoes. Similarly, the antioxidant activity measured by the DPPH method in yellow tree tomatoes ranged from 4.9 to 31.4 µmol ascorbic acid equivalents (AAE)/g DW. Purple tree tomatoes ranged from 7.3 to 47.1 µmol AAE/g DW [2]. Tart cherry showed antioxidant activity of 7.1 µmol TE/g fresh weight (FW) in April and 5.8 µmol TE/g FW in July using the ABTS method [38]. Purple taxo (*P. mollissima*) showed values of 70.0 µmol TE/100 g FW, 20,754.9 µmol TE/100 g FW, and 60,843.1 µmol TE/100 g FW using the DPPH method [51]. Finally, chamburo (*V. pubescens*) showed a variation from the non-detectable limit to 20.6 mM TE/100 g by the DPPH method [47].

### 3.4. Statistical Analyses

**Figure 3:**
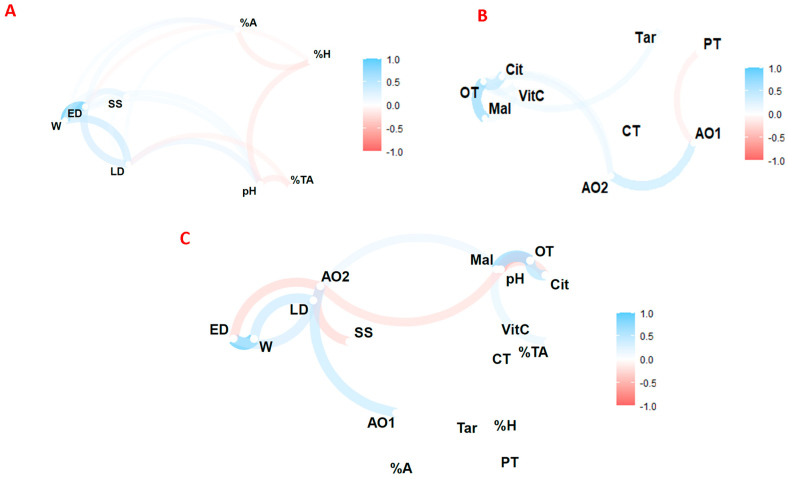
Pearson correlation coefficients of commercial quality (**A**), bioactive compounds (**B**), and all studied variables (**C**). Note: W: weight; ED: equatorial diameter; LD: longitudinal diameter; SS: soluble solid; %TA, total titratable acidity; %H: humidity; %A: ash; VitC: vitamin C; Cit: citric acid; Tar: tartaric acid; Mal: Malic acid; OT: total organic acid; CT: total carotenoids; PT: total phenolics; AO1: antioxidant activity by ABTS; AO2: antioxidant activity by DPPH.

Pearson linear correlation coefficients are shown in Figure 3 at the 99% confidence level. Figure 3A shows the statistical analysis of the commercial quality parameters. Figure 3B shows the statistical analysis of bioactive compounds. Figure 3C shows the overall statistical analysis. The results showed correlations that have been widely investigated in other studies, such as an inverse correlation between soluble solids and pH, and a direct correlation between weight and diameter, total phenolics and antioxidant activity, total carotenoids, and colour intensity. In addition, significant positive correlations were observed between weight and longitudinal diameter (0.5) and between weight and equatorial diameter (0.9). A positive correlation was also observed between titratable acidity and malic acid (0.4), citric acid and total organic acids (0.6), and malic acid and total organic acids (0.7). Significant positive correlations were also found between antioxidant activity measured by the ABTS and DPPH methods (0.5). On the other hand, significant negative correlations were found between pH and citric acid (−0.4), pH and total organic acids (−0.5), soluble solids, and antioxidant activity measured by DPPH (−0.4).

**Figure 4:**
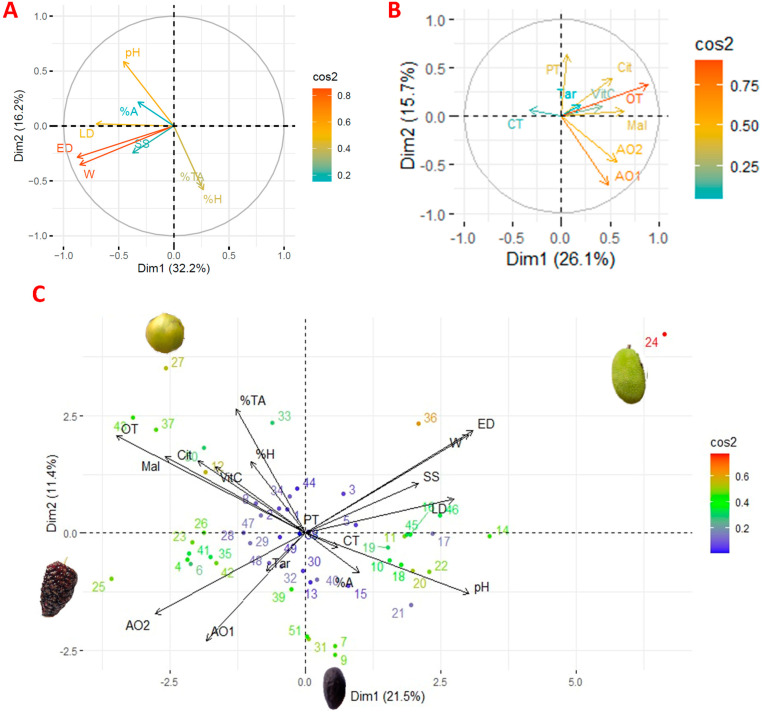
Exploratory multivariate analysis using PCA ((**A**) commercial quality; (**B**) bioactive compounds; (**C**) all studied variables). Note: W: weight; ED: equatorial diameter; LD: longitudinal diameter; SS: soluble solid; %TA, total titratable acidity; %H: humidity; %A: ash; VitC: vitamin C; Cit: citric acid; Tar: tartaric acid; Mal: Malic acid; OT: total organic acid; CT: total carotenoids; PT: total phenolics; AO1: antioxidant activity by ABTS; AO2: antioxidant activity by DPPH.

The exploratory multivariate analysis using PCA is shown in Figure 4. Figure 4A shows the PCA of the commercial quality parameters. Figure 4B shows the PCA of bioactive compounds. Figure 4C shows the overall PCA. The results showed that the characteristics of each fruit are particulate; however, specific groups were mainly concentrated under the bioactivity characteristics, while others were placed under the commercial quality characteristics. It is important to note that some species, such as jackfruit (*A. heterophyllus*), tree blackberry (M. alba), and arazá (*E. stipitata*), move away from the cloud of dots representing the species under study, indicating distinctive characteristics that are common to the samples.

As for the PCA (Figure 4), it is observed that the Principal Component 1 explains 21.5% of the variability of the variables studied, while the Principal Component 2 explains 11.4%. It is highlighted that size, weight, and pH contribute positively to principal component 1, while antioxidant activity contributes negatively. On the other hand, titratable acidity, malic acid, and organic acids contribute positively to principal component 2.

Species such as ungurahua (*O. bataua*), copal (*D. peruviana*), momórdica (*M. charantia*), capulí (*P. salicifolia*), and uva de monte (*P. cecropiifolia*) show similarities in their characteristics. It has also been observed that tuna (O. ficus-indica), guava de bejuco (*I. edulis*), guava (*I. insignis*), zapote (*M. cordata*), cacao red (*T. cacao*), smooth-skinned cacao (*T. cacao*), and mamey (*P. sapota*) also share specific characteristics.

## 4. Conclusions

Fruits are an important source of bioactive compounds; however, more information about minor tropical fruits is needed. In this sense, this study aimed to evaluate the bioactive compounds and antioxidant capacity of fifty-one minor tropical fruits consumed in Ecuador. The results showed high values in different parameters, highlighting the uniqueness of each species. White chayote (*Sechium edulis*) was characterised by high pH (8.2), mamey (*Pouteria sapota*) by soluble solids (28.6 °Brix), arazá (*Eugenia stipitata*) by total titratable acidity (4.6%), red ovo (*Spondias purpurea* red) for high moisture content (96.5%), achotillo (*Nephelium lappaceum*) for ash (20.1%), cereza agria (*Dovvalis hebecarpa*) for vitamin C (768.2 mg/100 g DW), yellow taxo (*Passiflora tripartita*) for total organic acids (37.2 g/100 g DW), momórdica (*Momordica charantia*) for total carotenoids (487.0 mg/100 g DW), achotillo (*Nephelium lappaceum*) for total phenolic compounds (535.4 mg/g DW), uva de monte (*Pourouma cecropiifolia*) for antioxidant activity according to the ABTS method (161.4 µmol ET/g DW), and mora de árbol (*Morus alba*) for antioxidant activity according to the DPPH method (80.5 µmol AAE/g DW). It is particularly relevant to highlight the significant presence of vitamin C in several tropical fruits, making them valuable sources of this essential vitamin for the human organism. In addition, the variability in the concentration of organic acids, carotenoids responsible for the wide range of colours, and phenolic compounds add value to these fruits. However, it is essential to note that the extraction of bioactive compounds such as carotenoids and phenolic compounds is influenced by many factors, particularly the type of solvent used. In this context, it has been shown that a combination of solvents such as methanol, acetone, and dichloromethane in specific ratios (1:1:2), together with an extraction time of 2 min, represents one of the optimal conditions for the extraction of carotenoids. Similarly, a solution consisting of 80% methanol acidified with 0.1% hydrochloric acid and an extraction time of 3 min has proved highly effective for extracting phenolic compounds. These conditions allow for maximising the extraction yield of the above bioactive compounds, thus increasing their viability and usefulness in various applications of interest for health and nutrition.

## Figures and Tables

**Table 1:** Location of points of sale of the different non-traditional tropical fruits studied.

N°	Family	Scientific Name	Common Name	Sampling Location	Provincia	Reference Image
1	Anacardiaceae	*Spondias purpurea* L. red	Red ovo	Sangolquí market	Pichincha	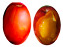
2		*Spondias purpurea* L. yellow	Yellow ovo	Ilumán market	Imbabura	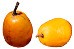
3	Apoxynaceae	*Lacmellea lactescens* (Kuhlm.) Markgr.	Chicle	Pedro Vicente Maldonado market	Pichincha	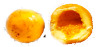
4	Arecaceae	*Bactris concinna* Mart.	Uva de chonta	Tena market	Napo	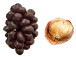
5		*Bactris gasipaes* Kunth	Chonta naranja	Plantation	Sucumbíos	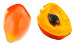
6		*Mauritia flexuosa* L.f.	Moriche negro	Bomboiza market	Morona Santiago	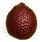
7		*Oenocarpus bataua* Mart.	Ungurahua	Bomboiza market	Morona Santiago	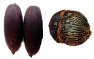
8		*Salacca zalacca* (Gaertn.) Voss	Salak	Nanegalito market	Pichincha	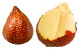
9	Burseraceae	*Dacryodes peruviana* (Loes.) H.J.Lam	Copal	Bomboiza market	Morona Santiago	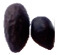
10	Cactaceae	*Opuntia ficus-indica* (L.) Mill.	Tuna	Guamaní market	Pichincha	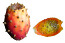
11		*Selenicereus megalanthus* (K.Schum. ex Vaupel) Moran	Pitahaya	Baños market	Tungurahua	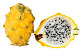
12	Caricaceae	*Vasconcellea pubescens* A. DC.	Chamburo	Saraguro market	Loja	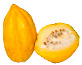
13	Curcubitaceae	*Momordica charantia* L.	Momórdica	Plantation	Santo Domingo de los Tsáchilas	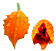
14		*Sicana odorifera* Naudin	Calabaza	Puyo market	Pastaza	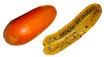
15		*Sicyos edulis* Jacq.	Chayote blanco	Puyo market	Pastaza	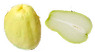
16	Ebenaceae	*Diospyros kaki* L.f.	Caqui	Mayorista market	Pichincha	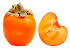
17	Fabaceae	*Inga edulis* Mart.	Guaba de bejuco	Santa Clara market	Pichincha	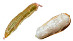
18		*Inga insignis* Kunth	Guaba	Sangolquí market	Pichincha	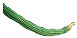
19	Malvaceae	*Herrania nitida* (Poepp.) R.E.Schult.	Rough skin cacao	Majua market	Esmeraldas	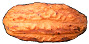
20		*Matisia cordata* Bonpl.	Zapote	Mayorista market	Pichincha	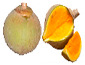
21		*Theobroma cacao* L. red	Cacao rojo	Plantation	Santo Domingo de los Tsáchilas	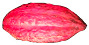
22		*Theobroma cacao* L. yellow	Smooth-skinned cacao	Bomboiza market	Morona Santiago	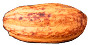
23	Melastomataceae	*Miconia crocea* Naudin	Kulkas	Otavalo market	Ibarra	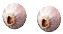
24	Moraceae	*Artocarpus heterophyllus* Lam.	Jackfruit	Santa Clara market	Pichincha	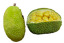
25		*Morus alba* L.	Tree blackberry	Plantation	Pichincha	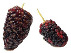
26	Myrtaceae	*Acca sellowiana* (O. Berg) Burret	Feijoa	Plantation	Tungurahua	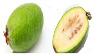
27	Myrtaceae	*Eugenia stipitata* McVaugh	Arazá	Sucumbíos market	Sucumbíos	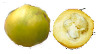
28		*Psidium guajava* L.	Guayaba roja	Plantation	Tungurahua	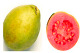
29		*Psidium guineense* Sw.	Guayaba amarilla	Plantation	Tungurahua	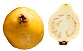
30		*Syzygium jambos* (L.) Alston red	Red pomarrosa	El Coca market	Orellana	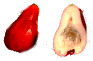
31		*Syzygium jambos* (L.) Alston yellow	Yellow pomarrosa	La Unión market	Sucumbíos	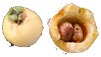
32	Oxalidaceae	*Averrhoa carambola* L.	Fruta china	Cotocollao market	Pichincha	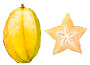
33	Passifloraceae	*Passiflora edulis* Sims	Granadilla de monte	Tumbaco market	Pichincha	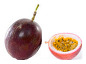
34		*Passiflora hybrid*	Maracuyá-badea	Puyo market	Pastaza	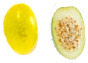
35		*Passiflora mollissima* L.H.Bailey	Purple taxo	Plantation	Tungurahua	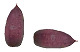
36		*Passiflora quadrangularis* L.	Badea	Plantation	Santo Domingo de los Tsáchilas	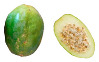
37		*Passiflora tripartita* Breiter	Yellow taxo	Plantation	Pichincha	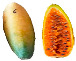
38	Rosaceae	*Cydonia oblonga* Mill.	Membrillo	Santa Clara market	Pichincha	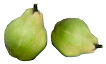
39	Rosaceae	*Prunus persica* (L.) Batsch	Abridores	Santa Clara market	Pichincha	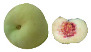
40		*Prunus salicifolia* Kunth	Capulí	Sangolquí market	Pichincha	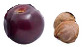
41		*Rubus niveus* Thunb.	Wild blackberry	Plantation	Pichincha	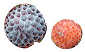
42		*Rubus rosifolius* Sm.	Raspberry	Plantation	Pichincha	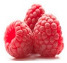
43	Salicaceae	*Dovyalis hebecarpa* (Gardner) Warb.	Cereza agria	Plantation	Pichincha	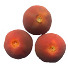
44	Sapindaceae	*Nephelium lappaceum* L.	Achotillo	Sangolquí market	Pichincha	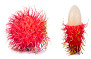
45	Sapotaceae	*Pouteria caimito* Radlk.	Caimito amarillo	La Unión market	Sucumbíos	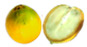
46		*Pouteria sapota* (Jacq.) H.E.Moore & Stearn	Mamey	La Unión market	Sucumbíos	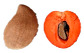
47	Solanaceae	*Physalis peruviana* L.	Uvilla	Tumbaco market	Pichincha	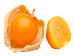
48		*Solanum betaceum* Cav. purple	Tomate de árbol morado	Plantation	Tungurahua	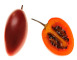
49		*Solanum betaceum* Cav. yellow	Tomate de árbol amarillo	Plantation	Tungurahua	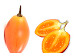
50		*Solanum sessiliflorum* Dunal	Cocona	Macas market	Morona Santiago	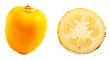
51	Urticaceae	*Pourouma cecropiifolia* Mart.	Uva de monte	El Coca market	Orellana	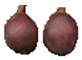

**Table 2:** Average commercial quality of the different non-traditional tropical fruits studied.

Family	Scientific Name	Weight (g)			LD (cm)			ED (cm)			pH			SS (°Brix)			TA (%)			Humidity (%)			Ash (%)		
Anacardiaceae	*S. purpurea* red	19.7	±	1.8 ^abc^	2.9	±	0.2 ^abcde^	3.6	±	0.2 ^efghijk^	3.3	±	0.2 ^cde^	11.9	±	1.2 ^ijklmno^	0.1	±	0.0 ^mnopq^	96.5	±	37.7 ^tuv^	0.6	±	0.3 ^abcdef^
	*S. purpurea* yellow	7.9	±	1.5 ^a^	4.8	±	0.6 ^efghij^	3.3	±	0.6 ^defghij^	3.1	±	0.5 ^bc^	17.1	±	1.9 ^pqrst^	1.0	±	0.3 ^mnopq^	76.0	±	5.1 ^efghi^	0.4	±	0.1 ^abc^
Apoxynaceae	*L. lactescens*	13.3	±	3.8 ^ab^	3.2	±	0.4 ^bcdef^	2.9	±	0.4 ^bcdefghi^	4.0	±	0.4 ^ghijkl^	17.2	±	1.3 ^pqrst^	3.0	±	0.3 ^u^	71.7	±	1.0 ^def^	0.7	±	0.2 ^abcdefg^
Arecaceae	*B. concinna*	12.0	±	1.0 ^ab^	3.8	±	0.4 ^cdefgh^	2.6	±	0.2 ^bcdefgh^	3.8	±	0.2 ^fghi^	9.7	±	0.7 ^cdefghijkl^	0.8	±	0.2 ^hjklmno^	83.5	±	2.0 ^opqr^	0.9	±	0.2 ^fghij^
	*B. gasipaes*	46.0	±	6.8 ^abcd^	5.2	±	0.2 ^fghijkl^	4.1	±	0.2 ^ghijklmno^	6.7	±	0.3 ^uv^	6.7	±	0.1 ^abcdefghi^	2.9	±	0.3 ^u^	44.9	±	0.9 ^b^	0.9	±	0.1 ^fghij^
	*M. flexuosa*	39.4	±	2.7 ^abcd^	4.4	±	1.5 ^fghijk^	3.7	±	1.1 ^efghijkl^	4.6	±	0.7 ^hijklm^	4.7	±	0.2 ^abcd^	0.7	±	0.1 ^hijklmno^	68.1	±	0.7 ^cd^	0.9	±	0.2 ^fghij^
	*O. bataua*	12.1	±	1.2 ^ab^	3.3	±	0.2 ^bcdef^	2.3	±	0.1 ^bcdef^	7.3	±	0.1 ^w^	2.2	±	0.3 ^a^	0.3	±	0.1 ^abcde^	42.7	±	0.0 ^b^	1.3	±	0.0 ^klmno^
	*S. zalacca*	56.0	±	7.3 ^abcd^	6.0	±	0.6 ^ijklmn^	5.1	±	0.3 ^klmnop^	4.2	±	0.2 ^hijklmn^	14.5	±	1.1 ^lmnop^	0.6	±	0.1 ^efghijkl^	97.1	±	0.2 ^uv^	0.7	±	0.2 ^abcdefg^
Burseraceae	*D. peruviana*	12.6	±	0.9 ^ab^	3.5	±	0.1 ^cdef^	2.3	±	0.1 ^abcde^	5.7	±	0.3 ^t^	3.9	±	1.0 ^ab^	0.5	±	0.0 ^cdefghij^	33.6	±	0.7 ^a^	7.6	±	1.0 ^w^
Cactaceae	*O. ficus-indica*	132.8	±	27.2 ^abcde^	8.2	±	1.1 ^op^	5.4	±	0.3 ^nopqr^	6.6	±	1.2 ^uv^	11.1	±	1.8 ^fghijklmn^	0.3	±	0.1 ^abcde^	85.6	±	2.9 ^mnopqr^	0.4	±	0.0 ^ab^
	*S. megalanthus*	292.0	±	7.1 ^efg^	11.1	±	0.6 ^qr^	7.3	±	0.6 ^tuvw^	5.3	±	0.3 ^rst^	15.5	±	0.9 ^nopqr^	0.2	±	0.0 ^abc^	75.1	±	2.6 ^efghi^	0.4	±	0.1 ^ab^
Caricaceae	*V. pubescens*	144.7	±	38.8 ^abcde^	7.6	±	1.3 ^nop^	5.0	±	1.2 ^klmnopq^	4.6	±	1.0 ^nop^	6.6	±	0.6 ^abcdefgh^	1.0	±	0.2 ^nopq^	93.0	±	0.1 ^stuv^	1.5	±	0.2 ^mnop^
Curcubitaceae	*M. charantia*	4.2	±	1.6 ^a^	4.3	±	1.7 ^defghij^	3.8	±	0.8 ^efghijklm^	4.0	±	0.0 ^ghijk^	6.4	±	0.2 ^abcdefg^	0.2	±	0.0 ^abcd^	93.4	±	0.3 ^stuv^	3.5	±	0.3 ^u^
	*S. odorifera*	2220.0	±	34.2 ^j^	36.5	±	0.0 ^u^	10.8	±	0.0 ^y^	6.7	±	0.0 ^v^	6.9	±	0.1 ^abcdefghij^	0.2	±	0.0 ^abcd^	88.3	±	0.1 ^ghijklmnopq^	1.5	±	0.3 ^nop^
	*S. edulis*	415.0	±	15.9 ^fgh^	11.8	±	0.9 ^r^	8.6	±	0.9 ^wx^	8.2	±	0.2 ^x^	4.6	±	0.5 ^abc^	0.1	±	0.0 ^a^	91.7	±	0.4 ^qrstu^	0.6	±	0.0 ^abcdefg^
Ebenaceae	*D. kaki*	153.7	±	3.9 ^abcde^	4.3	±	1.0 ^defghij^	6.4	±	1.0 ^qrstuv^	6.4	±	1.0 ^uv^	15.7	±	1.2 ^nopqr^	0.5	±	0.1 ^bcdefghi^	79.2	±	2.7 ^ghijklm^	0.8	±	0.2 ^defghi^
Fabaceae	*I. edulis*	480.0	±	92.9 ^ghi^	88.4	±	7.2 ^w^	3.7	±	1.1 ^efghijklm^	6.2	±	0.6 ^u^	11.3	±	1.2 ^ghijklmn^	0.1	±	0.0 ^a^	84.9	±	1.0 ^klmnop^	0.4	±	0.1 ^abcd^
	*I. insignis*	75.9	±	9.0 ^abcde^	18.4	±	2.4 ^s^	3.2	±	0.3 ^cdefghij^	6.5	±	0.3 ^uv^	12.1	±	1.3 ^jklmno^	0.1	±	0.0 ^a^	87.2	±	5.5 ^opqrs^	0.4	±	0.1 ^abycd^
Malvaceae	*H. nitida*	871.0	±	47.7 ^i^	19.7	±	0.3 ^t^	9.3	±	0.2 ^xy^	5.6	±	0.2 ^st^	19.6	±	2.0 ^abcdefghijk^	1.6	±	0.3 ^lmnop^	96.0	±	0.0 ^opqrstuv^	4.6	±	0.1 ^v^
	*M. cordata*	516.6	±	10.7 ^hi^	8.2	±	0.7 ^op^	7.7	±	0.8 ^vw^	7.5	±	0.8 ^w^	16.6	±	2.1 ^opqr^	0.6	±	0.3 ^efghijkl^	73.1	±	10.9 ^defg^	0.7	±	0.1 ^bcdefg^
	*T. cacao* red	694.0	±	87.0 ^fghi^	21.5	±	0.2 ^st^	8.8	±	0.1 ^qrstuv^	4.5	±	0.2 ^ijklmno^	15.4	±	3.1 ^mnopq^	0.6	±	0.2 ^defghijklm^	88.1	±	0.1 ^defghijk^	20.1	±	4.9 ^x^
	*T. cacao* yellow	1252.7	±	41.6 ^hi^	22.3	±	0.6 ^sst^	11.8	±	0.4 ^pqrstuv^	4.3	±	0.1 ^hijklmno^	18.4	±	4.0 ^rst^	0.7	±	0.0 ^cdefghijklm^	88.3	±	1.1 ^cdefghijk^	0.9	±	0.0 ^abcdefg^
Melastomataceae	*M. crocea*	0.4	±	0.1 ^a^	1.0	±	0.2 ^a^	0.7	±	0.2 ^a^	4.1	±	0.0 ^hijklmn^	8.3	±	1.3 ^bcdefghijk^	0.8	±	0.2 ^ijklmno^	84.1	±	0.2 ^klmnop^	0.6	±	0.3 ^abcdef^
Moraceae	*A. heterophyllus*	9117.2	±	143.0 ^l^	43.5	±	3.6 ^v^	27.5	±	7.6 ^z^	6.2	±	0.7 ^u^	19.4	±	1.2 ^rst^	1.1	±	0.2 ^pqrs^	74.2	±	3.0 ^defgh^	3.3	±	0.3 ^u^
	*M. alba*	3.5	±	0.6 ^a^	3.6	±	0.2 ^cdefg^	2.5	±	0.2 ^bcdefgh^	1.0	±	0.0 ^a^	6.0	±	1.3 ^abcdef^	0.4	±	0.2 ^abcdefg^	92.0	±	1.2 ^rstu^	0.7	±	0.2 ^bcdefg^
Myrtaceae	*A. sellowiana*	45.3	±	6.3 ^abcd^	6.2	±	0.7 ^jklmn^	2.5	±	0.2 ^bcdefgh^	3.9	±	0.6 ^fghij^	9.9	±	0.9 ^defghijklm^	0.7	±	0.2 ^fghijklmn^	86.1	±	0.3 ^nopqr^	1.2	±	0.5 ^jklmn^
	*E. stipitata*	98.7	±	35.3 ^abcde^	5.0	±	0.9 ^fghijk^	5.2	±	1.0 ^lmnopqr^	4.0	±	0.4 ^ghijkl^	5.3	±	1.9 ^abcde^	4.6	±	1.8 ^w^	94.1	±	7.9 ^tuv^	0.3	±	0.0 ^a^
	*P. guajava*	37.8	±	10.0 ^abcd^	4.7	±	0.6 ^efghij^	4.0	±	0.5 ^ghijklmno^	4.5	±	0.2 ^mnop^	10.1	±	0.8 ^efghijklm^	0.8	±	0.1 ^jklmno^	73.6	±	2.5 ^defgh^	2.3	±	0.3 ^rst^
	*P. guineense*	60.7	±	17.1 ^abce^	5.7	±	0.9 ^hijklmn^	5.1	±	0.4 ^klmnopq^	5.5	±	0.0 ^st^	7.5	±	0.0 ^bcdefghijk^	0.5	±	0.0 ^cdefghij^	73.6	±	1.3 ^defgh^	2.3	±	0.2 ^rst^
	*S. jambos* red	75.8	±	14.0 ^abcde^	6.1	±	0.8 ^ijklmn^	5.7	±	0.9 ^opqrst^	4.4	±	0.4 ^klmno^	9.1	±	3.1 ^bcdefghijkl^	0.2	±	0.1 ^abcde^	91.3	±	0.^pqrst^	0.4	±	0.0 ^ab^
	*S. jambos* yellow	24.2	±	2.1 ^abc^	4.6	±	0.3 ^efghij^	4.0	±	0.2 ^fghijklmn^	6.6	±	0.2 ^uv^	9.4	±	0.9 ^cdefghijkl^	0.4	±	0.2 ^abcdefg^	90.1	±	0.7 ^rstu^	0.6	±	0.0 ^abcdef^
Oxalidaceae	*A. carambola*	52.1	±	9.5 ^abcd^	6.9	±	0.7 ^klmno^	4.7	±	0.7 ^jklmnop^	4.8	±	0.1 ^pqr^	5.3	±	0.2 ^abcde^	0.7	±	0.1 ^fghijklmn^	92.0	±	0.3 ^rstu^	0.7	±	0.1 ^cdefgh^
Passifloraceae	*P. edulis*	30.0	±	11.0 ^abcd^	4.4	±	0.5 ^efghij^	4.1	±	0.5 ^ghijklmno^	4.2	±	1.3 ^hijklmn^	22.0	±	3.4 ^t^	1.4	±	0.1 ^rst^	94.1	±	0.3 ^tuv^	2.3	±	0.5 ^st^
	*P. hybrid*	246.0	±	18.8 ^def^	11.3	±	0.3 ^r^	7.1	±	0.1 ^stuvw^	4.2	±	0.2 ^hijklmn^	12.7	±	2.8 ^klmnop^	0.9	±	0.0 ^klmnop^	94.1	±	0.7 ^tuv^	1.1	±	0.1 ^ijklm^
	*P. mollissima*	57.2	±	6.7 ^abcd^	7.6	±	0.3 ^nop^	3.8	±	0.2 ^efghijklm^	4.5	±	0.4 ^lmnof^	8.5	±	1.0 ^bcdefghijk^	0.4	±	0.1 ^abcdefgh^	76.4	±	3.1 ^efghi^	2.4	±	0.3 ^t^
	*P. quadrangularis*	2470.0	±	27.0 ^k^	22.9	±	6.9 ^t^	19.7	±	5.2 ^z^	3.7	±	0.8 ^efgh^	11.9	±	1.9 ^jklmno^	1.2	±	0.4 ^qrs^	78.8	±	5.2 ^ghijkl^	2.2	±	0.1 ^st^
	*P. tripartita*	73.0	±	10.6 ^abcde^	3.6	±	0.2 ^cdefg^	10.7	±	0.7 ^y^	3.2	±	0.2 ^bcd^	10.9	±	0.0 ^fghijklmn^	0.4	±	0.0 ^abcdefg^	81.1	±	2.1 ^ijklmno^	1.8	±	0.1 ^pq^
Rosaceae	*C. oblonga*	102.9	±	16.5 ^abcde^	5.5	±	0.8 ^ghijklm^	6.0	±	0.7 ^pqrstu^	3.2	±	0.1 ^bcd^	14.5	±	0.7 ^lmnop^	0.7	±	0.3 ^fghijklmn^	71.0	±	4.2 ^de^	0.5	±	0.1 ^abcde^
	*P. persica*	48.6	±	4.3 ^abcd^	4.5	±	0.3 ^efghij^	4.2	±	0.2 ^hijklmno^	4.8	±	0.2 ^opq^	12.5	±	1.1 ^klmnop^	0.5	±	0.2 ^defghijk^	85.2	±	1.6 ^klmnop^	2.5	±	0.8 ^t^
	*P. salicifolia*	2.3	±	0.5 ^a^	1.4	±	0.1 ^ab^	1.5	±	0.1 ^abc^	5.1	±	0.0 ^qrs^	19.2	±	1.2 ^rst^	0.4	±	0.1 ^abcdefgh^	77.6	±	2.7 ^fghij^	0.7	±	0.3 ^bcdefg^
	*R. niveus*	1.6	±	0.4 ^a^	1.0	±	0.1 ^a^	1.3	±	0.2 ^ab^	1.0	±	0.0 ^a^	11.4	±	2.0 ^ghijklmn^	1.6	±	0.7 ^t^	84.1	±	1.9 ^klmnop^	0.7	±	0.2 ^bcdefg^
	*R. rosifolius*	1.7	±	0.3 ^a^	1.7	±	0.2 ^abc^	1.6	±	0.2 ^abc^	3.9	±	0.2 ^fghij^	5.0	±	0.7 ^abcde^	0.7	±	0.3 ^ghijklmn^	83.9	±	1.0 ^jklmnop^	0.6	±	0.0 ^abcdef^
Salicaceae	*D. hebecarpa*	7.6	±	0.6 ^a^	1.9	±	0.1 ^abc^	2.5	±	0.1 ^bcdefg^	2.7	±	0.1 ^b^	11.6	±	0.7 ^hijklmno^	3.2	±	0.6 ^u^	85.1	±	1.1 ^lmnop^	1.9	±	0.5 ^pqr^
Sapindaceae	*N. lappaceum*	20.5	±	2.5 ^abc^	4.1	±	0.2 ^defghi^	3.2	±	0.2 ^cdefghij^	4.3	±	0.5 ^jklmn^	15.0	±	1.2 ^mnopq^	0.4	±	0.2 ^abcdef^	78.5	±	1.0 ^ghijk^	0.3	±	0.2 ^ab^
Sapotaceae	*P. caimito*	238.7	±	52.4 ^cdef^	7.2	±	0.6 ^mnop^	7.6	±	0.6 ^uvw^	5.3	±	0.3 ^rst^	20.6	±	0.8 ^st^	1.5	±	0.2 ^st^	88.3	±	0.4 ^jklmno^	1.9	±	0.4 ^qrs^
	*P. sapota*	232.2	±	59.2 ^bcdef^	9.1	±	1.0 ^pq^	6.8	±	0.7 ^rstuv^	6.3	±	0.2 ^uv^	28.6	±	3.4 ^u^	0.6	±	0.1 ^fghijklm^	62.9	±	1.4 ^c^	1.0	±	0.2 ^ghijk^
Solanaceae	*P. peruviana*	5.3	±	2.2 ^a^	1.9	±	0.2 ^abc^	1.9	±	0.2 ^abcd^	4.5	±	0.0 ^mnop^	12.0	±	0.0 ^jklmno^	1.1	±	0.0 ^pqr^	81.0	±	0.3 ^ijklmno^	1.4	±	0.1 ^lmno^
	*S. betaceum* purple	101.7	±	21.9 ^abcde^	7.0	±	1.1 ^lmno^	5.2	±	0.7 ^mnopqr^	3.5	±	0.5 ^defg^	11.5	±	1.9 ^ghijklmn^	1.0	±	0.6 ^lmnop^	79.8	±	7.1 ^hijklmn^	3.5	±	0.8 u
	*S. betaceum* yellow	120.3	±	27.4 ^abcde^	7.4	±	0.3 ^mnop^	5.6	±	0.5 ^nopqrs^	3.5	±	0.1 ^cdef^	10.0	±	0.8 ^efghijklm^	1.1	±	0.4 ^pqr^	79.6	±	0.3 ^hijklm^	1.7	±	0.9 ^opq^
	*S. sessiliflorum*	40.2	±	5.8 ^abcd^	3.7	±	0.1 ^cdefg^	4.3	±	0.4 ^ijklmno^	3.8	±	0.0 ^efgh^	6.0	±	0.0 ^abcdef^	3.8	±	0.2 ^v^	94.1	±	0.3 ^tuv^	1.0	±	0.0 ^ghijk^
Urticaceae	*P. cecropiifolia*	7.7	±	1.0 ^a^	2.4	±	0.1 ^abcd^	2.4	±	0.1 ^bcdef^	5.7	±	0.1 ^t^	15.5	±	2.2 ^defghijk^	0.6	±	0.0 ^defghijk^	98.6	±	0.1 ^v^	0.8	±	0.0 ^efghi^

Note: The lower case letters next to the standard deviation indicate the separation of the mean values of the tropical fruits studied at a 95% confidence level. LD, longitudinal diameter; ED, equatorial diameter; SS, soluble solids; TA, total titratable acid.

**Table 3:** The different minor tropical fruits studied average vitamin C, organic acid, and antioxidant activity levels.

Family	Scientific Name	Vitamin C (mg/100 g DW)			Organic Acid (g/100 g DW)												Antioxidant Activity					
					Citric Acid			Malic Acid			Tartaric Acid			Total Organic Acid			µmol TE/g DW (ABTS)			µmol AAE/g DW (DPPH)		
Anacardiaceae	*S. purpurea* red	103.8	±	10.1	0.7	±	0.1 ^ab^	8.9	±	0.3 ^fghi^	2.5	±	0.0 ^abcde^	12.0	±	0.2 ^abcdefgh^	19.2	±	1.0 ^abcdef^	9.1	±	1.9 ^fghij^
	*S. purpurea* yellow	8.8	±	1.3	0.3	±	0.0 ^ab^	10.5	±	0.0 ^hij^	1.6	±	0.1 ^abc^	12.4	±	0.1 ^abcdefghi^	10.4	±	0.7 ^abcd^	31.9	±	0.4 ^rst^
Apoxynaceae	*L. lactescens*	8.1	±	0.4	6.1	±	0.2 ^bcdef^	0.8	±	0.0 ^a^	2.1	±	0.0 ^abcde^	8.9	±	0.2 ^abcdef^	10.2	±	1.6 ^abcd^	2.0	±	0.3 ^a^
Arecaceae	*B. concinna*	nd			2.9	±	0.0 ^abc^	13.7	±	0.0 ^jk^	7.8	±	0.0 ^fg^	24.3	±	0.0 ^jklm^	108.3	±	0.9 ^mnop^	33.6	±	0.0 ^st^
	*B. gasipaes*	13.8	±	1.7	0.2	±	0.0 ^ab^	0.6	±	0.0 ^a^	3.9	±	0.0 ^abcdef^	4.7	±	0.0 ^ab^	7.7	±	1.3 ^abc^	10.5	±	0.5 ^jk^
	*M. flexuosa*	nd			1.0	±	0.2 ^ab^	21.6	±	1.7 ^mn^	3.4	±	0.7 ^abcdef^	26.0	±	2.6 ^lm^	142.4	±	2.2 ^q^	38.8	±	4.3 ^u^
	*O. bataua*	2.7	±	0.1	0.7	±	0.1 ^ab^	2.7	±	0.6 ^abc^	1.1	±	0.1 ^abc^	4.5	±	0.7 ^ab^	72.7	±	1.0 ^hijk^	10.5	±	0.7 ^jk^
	*S. zalacca*	nd			1.4	±	0.1 ^abc^	20.5	±	1.5 ^lm^	2.7	±	0.4 ^abcde^	24.6	±	1.0 ^klm^	66.4	±	1.2 ^hi^	38.8	±	1.9 ^u^
Burseraceae	*D. peruviana*	nd			0.5	±	0.1 ^ab^	1.2	±	0.1 ^a^	0.6	±	0.0 ^ab^	2.3	±	0.6 ^a^	78.2	±	2.0 ^hijkl^	35.6	±	0.7 ^tu^
Cactaceae	*O. ficus-indica*	2.6	±	0.0	1.0	±	0.2 ^abc^	0.5	±	0.0 ^a^	3.6	±	0.2 ^abcdef^	5.0	±	0.1 ^abc^	25.7	±	4.7 ^bcdef^	4.8	±	0.9 ^abcde^
	*S. megalanthus*	1.3	±	0.0	0.9	±	0.0 ^abc^	0.8	±	0.1 ^a^	3.9	±	0.1 ^abcdef^	5.6	±	0.0 ^abcd^	13.2	±	2.4 ^abcd^	1.7	±	0.5 ^abc^
Caricaceae	*V. pubescens*	313.4	±	7.2	10.1	±	0.8 ^defg^	10.1	±	0.1 ^ghij^	3.9	±	0.0 ^abcdef^	24.1	±	0.8 ^jklm^	59.8	±	1.7 ^gh^	19.8	±	1.3 ^mn^
Curcubitaceae	*M. charantia*	nd			3.1	±	1.0 ^abc^	2.6	±	0.2 ^abc^	5.8	±	0.6 ^cdefg^	11.4	±	1.7 ^abcdefgh^	70.6	±	2.1 ^hij^	8.7	±	0.7 ^efghij^
	*S. odorifera*	nd			3.0	±	0.3 ^abc^	1.2	±	0.7 ^a^	1.1	±	0.1 ^abc^	5.4	±	0.2 ^abc^	12.4	±	0.6 ^abcd^	9.5	±	0.8 ^ghijk^
	*S. edulis*	27.7	±	1.8	0.7	±	0.1 ^ab^	7.2	±	0.1 ^defgh^	6.5	±	1.0 ^defg^	14.3	±	1.1 ^bcdefg^	22.6	±	0.9 ^abcdef^	33.7	±	0.2 ^st^
Ebenaceae	*D. kaki*	11.8	±	1.1	0.6	±	0.1 ^ab^	2.6	±	0.0 ^abc^	0.1	±	0.0 ^a^	3.2	±	0.1 ^a^	11.3	±	1.4 ^abcd^	4.2	±	1.3 ^abcdefg^
Fabaceae	*I. edulis*	6.6	±	0.3	6.9	±	0.4 ^cdef^	0.5	±	0.0 ^a^	3.7	±	0.1 ^abcdef^	11.1	±	0.4 ^abcdef^	78.6	±	2.5 ^hijkl^	7.3	±	1.7 ^defghij^
	*I. insignis*	16.9	±	2.6	0.5	±	0.1 ^ab^	0.7	±	0.0 ^a^	2.6	±	0.1 ^abcde^	3.9	±	0.2 ^a^	30.9	±	5.8 ^def^	1.5	±	0.4 ^abc^
Malvaceae	*H. nitida*	nd			3.6	±	0.1 ^abc^	0.9	±	0.2 ^a^	3.2	±	0.3 ^abcdef^	7.6	±	0.6 ^abcde^	92.0	±	21.3 ^nop^	20.1	±	3.8 ^lmn^
	*M. cordata*	25.5	±	3.2	0.9	±	0.2 ^ab^	1.2	±	0.1 ^ab^	2.9	±	0.0 ^abcde^	5.0	±	0.3 ^abc^	99.6	±	3.0 ^lmnop^	3.3	±	0.0 ^abcdef^
	*T. cacao* red	nd			4.6	±	0.4 ^abcd^	0.6	±	0.0 ^a^	2.7	±	0.1 ^abcde^	7.9	±	0.5 ^abcde^	111.2	±	0.4 ^nop^	18.1	±	2.6 ^lmn^
	*T. cacao* yellow	1.1	±	0.1	2.2	±	0.1 ^abc^	2.0	±	0.1 ^ab^	2.4	±	0.2 ^abcde^	6.6	±	1.5 ^abcd^	110.8	±	2.2 ^nop^	18.8	±	3.1 ^lmn^
Melastomataceae	*M. crocea*	11.2	±	1.3	1.4	±	0.3 ^abc^	16.9	±	0.5 ^kl^	1.8	±	0.3 ^abcde^	20.0	±	1.1 ^ghijklm^	94.7	±	21.3 ^klmno^	33.2	±	0.6 ^st^
Moraceae	*A. heterophyllus*	nd			0.7	±	0.1 ^ab^	3.2	±	0.1 ^abcd^	1.2	±	0.0 ^abc^	5.2	±	0.2 ^abc^	11.6	±	0.7 ^abcd^	2.4	±	0.1 ^abcd^
	*M. alba*	nd			13.3	±	0.5 ^gh^	2.5	±	0.0 ^abc^	4.4	±	0.4 ^abcdef^	20.1	±	0.9 ^ghijklm^	115.8	±	28.0 ^op^	80.5	±	3.9 ^x^
Myrtaceae	*A. sellowiana*	2.1	±	0.1	13.0	±	1.1 ^gh^	6.3	±	0.1 ^cdefg^	1.8	±	0.2 ^abcde^	21.1	±	1.0 ^ghijklm^	109.7	±	1.7 ^nop^	32.9	±	0.2 ^st^
	*E. stipitata*	9.8	±	0.0	3.6	±	0.5 ^abc^	25.0	±	2.9 ^n^	1.2	±	0.1 ^abc^	29.8	±	3.5 ^mn^	17.9	±	1.2 ^abcdef^	14.2	±	1.3 ^kl^
	*P. guajava*	577.1	±	0.4	5.1	±	0.1 ^abcde^	4.3	±	0.1 ^abcde^	2.1	±	0.3 ^abcd^	11.5	±	0.4 ^abcdefgh^	111.6	±	2.5 ^nop^	22.3	±	2.0 ^no^
	*P. guineense*	409.4	±	5.7	4.6	±	0.2 ^abcd^	7.2	±	0.0 ^defgh^	2.7	±	0.1 ^abcde^	14.5	±	0.1 ^cdefghijk^	94.4	±	2.2 ^klmno^	27.3	±	1.7 ^pqr^
	*S. jambos* red	nd			0.7	±	0.1 ^ab^	6.1	±	1.4 ^cdefg^	9.9	±	2.4 ^g^	16.7	±	4.6 ^efghijkl^	41.0	±	10.7 ^fg^	9.7	±	1.0 ^hijk^
	*S. jambos* yellow	2.8	±	0.2	0.7	±	0.2 ^ab^	3.3	±	0.2 ^abc^	7.0	±	1.0 ^efg^	10.9	±	1.9 ^abcdefg^	110.1	±	1.4^|nop^	31.1	±	0.0 ^rst^
Oxalidaceae	*A. carambola*	12.8	±	0.4	4.8	±	0.3 ^abcde^	3.0	±	0.2 ^abc^	2.5	±	0.0 ^abcde^	10.4	±	0.7 ^abcdefg^	115.1	±	1.0 ^op^	16.2	±	1.2 ^lm^
Passifloraceae	*P. edulis*	84.9	±	2.1	19.3	±	0.0 ^ij^	1.6	±	0.1 ^ab^	2.8	±	0.7 ^abcde^	23.8	±	0.7 ^jklm^	4.7	±	0.4 ^ab^	4.8	±	0.7 ^abcdefgh^
	*P. hybrid*	23.7	±	1.6	5.2	±	1.8 ^abcde^	12.7	±	4.2 ^ij^	4.6	±	0.6 ^abcdef^	22.5	±	8.3 ^ijklm^	63.8	±	1.3 ^h^	0.8	±	0.2 ^ab^
	*P. mollissima*	180.0	±	0.2	10.5	±	0.7 ^efg^	5.4	±	0.1 ^bcdef^	2.5	±	0.1 ^abcde^	18.4	±	1.9 ^fghijkl^	75.6	±	1.8 ^hijkl^	58.0	±	3.2 ^w^
	*P. quadrangularis*	62.8	±	2.7	5.3	±	0.5 ^abcde^	2.9	±	0.6 ^abc^	3.7	±	0.2 ^abcdef^	12.0	±	0.8 ^abcdefgh^	37.1	±	1.5 ^ef^	5.3	±	0.7 ^bcdefghi^
	*P. tripartita*	243.3	±	4.3	23.0	±	1.1 ^j^	8.8	±	0.5 ^fghi^	5.4	±	0.0 ^bcdef^	37.2	±	1.6 ^n^	91.3	±	0.5 ^jklmn^	19.5	±	3.7 ^lmn^
Rosaceae	*C. oblonga*	3.3	±	0.1	0.1	±	0.0 ^a^	8.5	±	0.2 ^fghi^	3.0	±	0.0 ^abcdef^	11.5	±	0.2 ^abcdefgh^	86.4	±	3.0 ^ijklm^	10.3	±	0.9 ^ijk^
	*P. persica*	nd			2.1	±	0.4 ^abc^	3.8	±	0.2 ^abcde^	3.6	±	0.3 ^abcdef^	9.6	±	0.0 ^abcdef^	110.6	±	0.9 ^nop^	30.1	±	0.2 ^qrs^
	*P. salicifolia*	9.9	±	1.3	4.9	±	0.5 ^abcde^	0.9	±	0.0 ^a^	0.6	±	0.2 ^a^	6.4	±	0.8 ^abcd^	73.9	±	1.3 ^hijkl^	33.0	±	0.3 ^st^
	*R. niveus*	nd			1.2	±	0.5 ^abc^	7.5	±	0.1 ^efgh^	2.3	±	0.2 ^abcde^	11.1	±	0.8 ^sbcdefgh^	110.6	±	1.9 ^nop^	45.0	±	1.4 ^v^
	*R. rosifolius*	26.6	±	1.3	10.1	±	1.1 ^efg^	2.6	±	0.1 ^abc^	2.3	±	0.2 ^abcde^	15.0	±	1.5 ^cdefghijk^	94.1	±	2.2 ^klmno^	33.1	±	1.4 ^st^
Salicaceae	*D. hebecarpa*	768.2	±	32.4	0.8	±	0.1 ^a^	17.3	±	1.6 ^kl^	2.3	±	0.1 ^abcde^	20.5	±	0.5 ^ghijklm^	92.4	±	2.7 ^jklmn^	22.5	±	1.6 ^nop^
Sapindaceae	*N. lappaceum*	73.4	±	1.6	11.2	±	1.5 ^defg^	2.4	±	0.1 ^abc^	2.0	±	0.2 ^abcde^	15.6	±	1.8 ^defghijk^	16.4	±	0.3 ^abcdef^	3.5	±	0.8 ^abcdefg^
Sapotaceae	*P. caimito*	24.3	±	1.3	0.3	±	0.1 ^a^	0.8	±	0.3 ^a^	4.9	±	1.4 ^bcdef^	6.1	±	1.7 ^abcd^	29.5	±	1.1 ^cdef^	2.6	±	0.4 ^abcde^
	*P. sapota*	56.6	±	1.0	0.8	±	0.0 ^a^	0.5	±	0.0 ^a^	1.8	±	0.2 ^abcde^	3.2	±	0.3 ^a^	18.3	±	1.1 ^abcde^	6.5	±	0.8 ^cdefghij^
Solanaceae	*P. peruviana*	124.3	±	2.4	9.7	±	0.9 ^efg^	1.8	±	0.1 ^ab^	2.8	±	0.0 ^abcde^	14.3	±	1.0 ^bcdefghij^	3.2	±	0.3 ^ab^	33.3	±	0.1 ^st^
	*S. betaceum* purple	83.2	±	4.2	5.3	±	0.9 ^abcde^	0.5	±	0.1 ^a^	7.6	±	0.8 ^efg^	13.4	±	1.7 ^bcdefghij^	117.3	±	0.4 ^p^	17.8	±	1.8 ^lmn^
	*S. betaceum* yellow	121.7	±	0.3	4.9	±	0.1 ^abcde^	0.5	±	0.0 ^a^	7.4	±	0.1 ^efg^	12.8	±	0.3 ^bcdefghij^	75.9	±	1.2 ^hijkl^	12.2	±	0.3 ^ijk^
	*S. sessiliflorum*	23.8	±	0.0	16.8	±	0.0 ^ij^	4.4	±	0.1 ^abcd^	1.7	±	0.0 ^abc^	22.8	±	0.1 ^jklm^	28.6	±	1.2 ^cdef^	26.0	±	0.7 ^opq^
Urticaceae	*P. cecropiifolia*	nd			4.8	±	0.1 ^abcde^	1.1	±	0.0 ^ab^	2.0	±	0.3 ^abcde^	7.9	±	0.3 ^abcde^	161.4	±	1.8 ^q^	30.2	±	3.1 ^qrs^

Note: The lower case letters next to the standard deviation indicate the separation of the mean values of the tropical fruits studied at a 95% confidence level. nd, not detectable.

**Table 4:** The different minor tropical fruits studied average carotenoids concentration.

Family	Scientific Name	α-Carotene			β-Carotene			β-Cryptoxanthin			Leteoxanthin			Lutein			Lycopene			Phytoene			Violaxanthin			Zeaxanthin			Zeinoxanthin			Total (mg/100 g DW)		
Anacardiaceae	*S. purpurea* red	2.9	±	0.1										0.6	±	0.0																3.6	±	0.1
	*S. purpurea* yellow	2.8	±	0.4										3.3	±	0.2							2.9	±	0.2				2.8	±	0.7	11.8	±	0.7
Apoxynaceae	*L. lactescens*	284.1	±	13.0																												284.1	±	13.0
Arecaceae	*B. concinna*				8.3	±	0.1																									8.3	±	0.1
	*B. gasipaes*	93.1	±	14.6	50.2	±	6.5	13.5	±	1.7	2.0	±	0.3	1.7	±	0.3	15.4	±	2.2										6.8	±	1.1	182.6	±	26.7
	*M. flexuosa*	5.5	±	0.1	19.9	±	1.9	7.0	±	0.8				1.3	±	0.1													3.7	±	0.3	37.4	±	1.4
	*O. bataua*																															nd		
	*S. zalacca*													0.8	±	0.0																0.8	±	0.0
Burseraceae	*D. peruviana*																															nd		
Cactaceae	*O. ficus-indica*													0.7	±	0.0							0.2	±	0.0				0.3	±	0.0	1.1	±	0.0
	*S. megalanthus*													0.8	±	0.1																0.8	±	0.1
Caricaceae	*V. pubescens*	13.6	±	1.2	33.6	±	0.1							12.9	±	0.1													1.1	±	0.1	61.4	±	1.2
Curcubitaceae	*M. charantia*													16.4	±	2.9	464.6	±	36.5				0.6	±	0.0				5.4	±	0.5	487.0	±	39.9
	*S. odorifera*				10.2	±	1.1													311.4	±	75.2										321.6	±	76.3
	*S. edulis*													0.9	±	0.0																0.9	±	0.0
Ebenaceae	*D. kaki*	13.6	±	0.2										0.9	±	0.1													1.0	±	0.0	15.5	±	0.3
Fabaceae	*I. edulis*				2.7	±	0.2																						0.8	±	0.1	3.4	±	0.3
	*I. insignis*																												0.8	±	0.0	0.8	±	0.0
Malvaceae	*H. nitida*																												0.9	±	0.1	0.9	±	0.1
	*M. cordata*	10.2	±	2.3	11.6	±	2.1	9.5	±	1.2				8.5	±	0.5	15.8	±	3.4										0.7	±	0.1	56.2	±	9.5
	*T. cacao* red				2.2	±	0.1																									2.2	±	0.1
	*T. cacao* yellow				0.8	±	0.1																						0.8	±	0.2	1.5	±	0.3
Melastomataceae	*M. crocea*													11.1	±	0.1													4.3	±	0.2	15.4	±	0.1
Moraceae	*A. heterophyllus*													0.5	±	0.0																0.5	±	0.0
	*M. alba*	19.8	±	3.9																									2.9	±	0.6	22.6	±	4.5
Myrtaceae	*A. sellowiana*				1.9	±	0.2																						6.4	±	0.4	8.2	±	0.3
	*E. stipitata*							9.8	±	2.0																			3.8	±	1.3	13.6	±	3.2
	*P. guajava*													1.1	±	0.0	59.5	±	5.9										1.2	±	0.0	61.8	±	5.9
	*P. guineense*													0.9	±	0.0													0.7	±	0.0	1.6	±	0.0
	*S. jambos* red													0.7	±	0.1																0.7	±	0.1
	*S. jambos* yellow													6.3	±	0.6													2.5	±	0.1	8.8	±	0.4
Oxalidaceae	*A. carambola*													1.7	±	0.0													3.6	±	0.0	5.3	±	0.0
Passifloraceae	*P. edulis*				5.0	±	0.7																									5.0	±	0.7
	*P. hybrid*	20.1	±	3.2				7.8	±	0.1	0.1	±	0.0																6.6	±	0.2	34.5	±	3.5
	*P. mollissima*				86.2	±	1.0							1.3	±	0.1													1.4	±	0.1	88.8	±	0.9
	*P. quadrangularis*													0.7	±	0.0													0.7	±	0.0	1.4	±	0.1
	*P. tripartita*													0.8	±	0.1							0.7	±	0.1				1.4	±	0.1	2.9	±	0.2
Rosaceae	*C. oblonga*							0.5	±	0.1																			0.9	±	0.1	1.4	±	0.2
	*P. persica*	2.5	±	0.0																												2.5	±	0.0
	*P. salicifolia*													13.8	±	1.0													1.6	±	0.0	15.4	±	1.1
	*R. niveus*													7.6	±	0.5													1.1	±	0.0	8.6	±	0.5
	*R. rosifolius*	4.8	±	0.1										2.3	±	0.5													1.3	±	0.3	8.4	±	0.2
Salicaceae	*D. hebecarpa*													9.8	±	1.4													1.7	±	0.4	11.5	±	1.9
Sapindaceae	*N. lappaceum*							1.6	±	0.1							2.4	±	0.3													4.1	±	0.4
Sapotaceae	*P. caimito*	4.7	±	0.2	1.7	±	0.0				0.5	±	0.0	0.6	±	0.0																7.6	±	0.3
	*P. sapota*				7.8	±	0.0	43.4	±	0.0				1.0	±	0.0													1.4	±	0.0	53.6	±	0.0
Solanaceae	*P. peruviana*													1.9	±	0.2										2.7	±	0.6	1.2	±	0.1	5.7	±	0.9
	*S. betaceum* purple				4.4	±	0.0	4.6	±	0.0				1.1	±	0.0																10.1	±	0.0
	*S. betaceum* yellow				0.8	±	0.1	2.6	±	0.4				0.3	±	0.0																3.7	±	0.5
	*S. sessiliflorum*				3.7	±	0.5																									3.7	±	0.5
Urticaceae	*P. cecropiifolia*																												90.9	±	8.4	90.9	±	8.4

**Table 5:** The different minor tropical fruits studied average phenolics concentration.

Family	Scientific Name	Chlorogenic Acid (mg/g)			Naringenin (mg/g)			Ferulic Acid (mg/g)			Quercetin (mg/g)			Siringic Acid (mg/g)			Routine (mg/g)			Cafeic Acid (mg/g)			*m*-Cumaric Acid (mg/g)			*p*-Coumaric Acid (mg/g)			*p*-Hydroxy Benzoic Acid (mg/g)			Gallic Acid (mg/g)			Total (mg/g)		
Anacardiaceae	*S. purpurea* red							0.3	±	0.1				0.2	±	0.1	17.6	±	4.4				1.8	±	0.6	0.1	±	0.0				0.2	±	0.0	20.2	±	5.2
	*S. purpurea* yellow	0.1	±	0.0	0.2	±	0.0	0.2	±	0.0																0.1	±	0.0				0.1	±	0.0	0.7	±	0.0
Apoxynaceae	*L. lactescens*				3.8	±	0.2				30.8	±	2.0							0.2	±	0.0	1.9	±	0.0				3.3	±	0.1	1.1	±	0.0	41.0	±	2.0
Arecaceae	*B. concinna*	0.8	±	0.0																0.7	±	0.2							3.4	±	0.0	7.9	±	0.2	12.7	±	0.3
	*B. gasipaes*	0.4	±	0.0				0.3	±	0.0				0.1	±	0.0				0.5	±	0.0							2.5	±	0.1	0.2	±	0.0	3.8	±	0.1
	*M. flexuosa*	0.1	±	0.0										0.2	±	0.0							0.5	±	0.0				2.6	±	0.1	0.1	±	0.0	3.5	±	0.1
	*O. bataua*	0.2	±	0.0				0.3	±	0.0										0.1	±	0.0	0.1	±	0.0				1.9	±	0.0				2.5	±	0.0
	*S. zalacca*	0.4	±	0.0							3.5	±	0.0	0.1	±	0.0				5.6	±	0.2							4.0	±	0.1	0.3	±	0.0	13.9	±	0.2
Burseraceae	*D. peruviana*	0.1	±	0.0	1.2	±	0.0													0.1	±	0.0							2.5	±	0.2				3.8	±	0.2
Cactaceae	*O. ficus-indica*	0.1	±	0.0	0.7	±	0.0	0.3	±	0.0				0.3	±	0.0				0.8	±	0.1				0.2	±	0.0	0.1	±	0.0	0.2	±	0.0	2.7	±	0.1
	*S. megalanthus*													1.6	±	0.4	50.5	±	8.5	0.7	±	0.2	0.6	±	0.1							0.1	±	0.0	53.4	±	9.3
Caricaceae	*V. pubescens* ^a^	0.1	±	0.0							15.9	±	0.3	0.4	±	0.0										0.1	±	0.0				0.6	±	0.0	78.9	±	7.3
Curcubitaceae	*M. charantia*	0.1	±	0.0	0.2	±	0.1	0.4	±	0.0	2.3	±	0.3	0.2	±	0.0							0.1	±	0.0				0.2	±	0.0	1.4	±	0.2	6.0	±	0.6
	*S. odorifera*	0.1	±	0.0				0.3	±	0.0				0.6	±	0.0				0.2	±	0.0							3.3	±	0.1	2.3	±	0.1	6.8	±	0.3
	*S. edulis* ^b^										91.1	±	8.5	0.1	±	0.0	237.3	±	5.2				0.9	±	0.1	0.3	±	0.0	2.5	±	0.1	0.5	±	0.1	487.1	±	13.5
Ebenaceae	*D. kaki*										18.3	±	0.0										0.6	±	0.0							0.2	±	0.0	19.1	±	0.1
Fabaceae	*I. edulis*	1.1	±	0.0	0.3	±	0.0													0.2	±	0.0	0.1	±	0.0							0.1	±	0.0	1.9	±	0.0
	*I. insignis*	1.4	±	0.1	0.2	±	0.0													0.2	±	0.0	1.0	±	0.1	0.1	±	0.0				0.3	±	0.0	3.1	±	0.2
Malvaceae	*H. nitida*	0.5	±	0.0	0.2	±	0.0							10.3	±	3.0				4.8	±	1.7				0.1	±	0.0	2.8	±	0.7	0.3	±	0.1	19.0	±	5.6
	*M. cordata*																			1.4	±	0.1				0.1	±	0.0				0.1	±	0.0	1.7	±	0.0
	*T. cacao* red	0.6	±	0.0										3.9	±	0.1				2.6	±	0.2	3.9	±	0.0	0.3	±	0.0	3.4	±	0.3	0.5	±	0.0	15.3	±	0.3
	*T. cacao* yellow	0.2	±	0.0	0.3	±	0.0	0.3	±	0.0				1.6	±	0.0				0.8	±	0.1	1.5	±	0.1	0.2	±	0.0	3.1	±	0.0	0.1	±	0.0	8.2	±	0.2
Melastomataceae	*M. crocea*	0.2	±	0.0				2.0	±	0.0										24.5	±	9.7	3.0	±	0.1	0.2	±	0.0	4.9	±	0.4	0.7	±	0.0	35.5	±	10.1
Moraceae	*A. heterophyllus*													0.1	±	0.0										0.1	±	0.0				0.2	±	0.0	0.3	±	0.0
	*M. alba*	40.0	±	0.3	0.4	±	0.0																			0.1	±	0.0				0.1	±	0.0	46.0	±	0.3
Myrtaceae	*A. sellowiana*				1.2	±	0.0	0.2	±	0.0										4.7	±	0.1	1.2	±	0.0	0.1	±	0.0				0.1	±	0.0	7.3	±	0.1
	*E. stipitata*	0.9	±	0.0	2.4	±	0.0	0.3	±	0.0										0.4	±	0.0	17.9	±	0.0							0.7	±	0.1	26.1	±	0.4
	*P. guajava*																												16.3	±	0.3	0.1	±	0.0	16.4	±	0.3
	*P. guineense*																												13.7	±	0.0	0.9	±	0.0	14.6	±	0.0
	*S. jambos* red										19.3	±	4.3																3.4	±	1.2	9.0	±	2.4	31.7	±	7.9
	*S. jambos* yellow				3.1	±	0.0	0.4	±	0.1	18.6	±	0.7	0.7	±	0.0				3.4	±	0.0	2.0	±	0.4	0.2	±	0.0	3.8	±	1.0	0.2	±	0.2	33.0	±	2.0
Oxalidaceae	*A. carambola*	0.5	±	0.0	2.8	±	0.2	0.3	±	0.0				0.1	±	0.0				1.3	±	0.0							2.2	±	0.0	0.1	±	0.0	7.3	±	0.1
Passifloraceae	*P. edulis*	0.6	±	0.2				87.0	±	2.5													7.7	±	2.3	0.6	±	0.2	17.4	±	4.9	0.4	±	0.1	113.7	±	33.1
	*P. hybrid*	0.7	±	0.1	0.3	±	0.0							0.5	±	0.0				2.2	±	0.0	0.6	±	0.0				3.0	±	0.3	1.0	±	0.0	8.3	±	0.2
	*P. mollissima*	0.1	±	0.0	1.9	±	0.1													1.5	±	0.0	2.6	±	0.1	0.1	±	0.0	11.6	±	0.2	0.2	±	0.0	18.1	±	0.4
	*P. quadrangularis*	0.8	±	0.2	0.3	±	0.0							0.6	±	0.1				1.2	±	0.3	0.2	±	0.0	0.2	±	0.0	0.2	±	0.0	0.3	±	0.1	8.3	±	2.2
	*P. tripartita*	0.7	±	0.1	1.0	±	0.0							0.2	±	0.0				1.6	±	0.0	1.2	±	0.0	0.4	±	0.0				0.3	±	0.0	5.3	±	0.1
Rosaceae	*C. oblonga*	1.3	±	0.0	2.6	±	0.0							0.6	±	0.0				2.3	±	0.1										0.1	±	0.0	7.2	±	0.2
	*P. persica*	4.4	±	1.2	0.3	±	0.1							0.1	±	0.0				30.7	±	8.6				0.1	±	0.0				0.2	±	0.0	35.9	±	1.0
	*P. salicifolia*	7.8	±	1.9	1.6	±	0.4							0.1	±	0.0				15.4	±	3.6	4.9	±	0.7	0.1	±	0.0				0.1	±	0.0	29.8	±	6.6
	*R. niveus*	13.4	±	1.3																						0.2	±	0.0	26.2	±	0.1	0.1	±	0.0	39.8	±	1.4
	*R. rosifolius*																						1.4	±	0.0				10.3	±	0.1	0.3	±	0.0	12.0	±	0.0
Salicaceae	*D. hebecarpa*	0.1	±	0.0										0.1	±	0.0				1.5	±	0.0	1.7	±	0.0	0.3	±	0.0	27.3	±	0.3	0.2	±	0.0	31.1	±	0.3
Sapindaceae	*N. lappaceum*	168.8	±	3.5				366.5	±	10.3																						0.1	±	0.0	535.4	±	13.9
Sapotaceae	*P. caimito*	0.1	±	0.0										0.6	±	0.0													3.2	±	0.5	1.7	±	0.1	5.6	±	0.6
	*P. sapota* ^c^													0.1	±	0.0													2.7	±	0.5	0.8	±	0.3	14.4	±	3.8
Solanaceae	*P. peruviana*	0.1	±	0.0	0.7	±	0.3							0.1	±	0.0	17.9	±	7.3	0.5	±	0.1	0.4	±	0.2	0.1	±	0.0				0.2	±	0.0	19.9	±	0.8
	*S. betaceum* purple							16.2	±	1.3										3.5	±	0.5													19.7	±	1.7
	*S. betaceum* yellow							14.9	±	1.4										5.6	±	0.1													20.5	±	1.2
	*S. sessiliflorum*	0.1	±	0.0	0.6	±	0.2							0.2	±	0.0				0.3	±	0.1	1.2	±	0.4	0.1	±	0.0	2.7	±	0.9	0.1	±	0.0	5.2	±	1.7
Urticaceae	*P. cecropiifolia*	5.9	±	0.6										0.8	±	0.0				8.7	±	0.3	4.4	±	0.3				2.5	±	0.1	0.3	±	0.0	22.7	±	0.4

^a^ Kaempferol (61.4 ± 7.6); ^b^ kaempferol (59.8 ± 1.0), quercetin glycoside (94.5 ± 0.5); ^c^ luteolin (10.8 ± 3.0).

## Data Availability

Data is contained within the article.

## Abbreviations

RRLC, rapid resolution liquid chromatography; DW, dry weight; TE, Trolox equivalent; AAE, Ascorbic acid equivalents; ABTS, 2,2′-azino-bis-(3-ethylbenzothiazoline-6-sulphonic acid; DPPH, 2,2-Diphenyl-1-picrylhydrazyl; PCA, principal component analysis; LD, longitudinal diameter; ED, equatorial diameter, SS, soluble solid; TA, total titratable acid; H-A, n-hexane: acetone (1:1); M-A-D, methanol: acetone; dichloromethane (1:1:2); M-T-W, methanol, trichloromethane, water (1:2:1); M, 100% methanol; A-E, acetone:ethanol (1:1); MA, 80% acidified methanol with 0.1% HCl; GAE, Gallic acid equivalent; W: weight; %H: humidity; %A: ash; VitC: vitamin C; Cit: citric acid; Tar: tartaric acid; Mal: Malic acid; OT: total organic acid; CT: total carotenoids; PT: total phenolics; AO1: antioxidant activity by ABTS; AO2: antioxidant activity by DPPH.
